# Heterogeneity of Treatment Effects of Laser Epilation on Pilonidal Disease Recurrence: A Randomized Clinical Trial

**DOI:** 10.1097/AS9.0000000000000488

**Published:** 2024-09-03

**Authors:** Katherine C. Bergus, Carley Lutz, Jennifer Cooper, Lindsey Asti, Lindsay Gil, Cory Criss, Katherine J. Deans, Peter C. Minneci

**Affiliations:** From the * Department of Pediatric Surgery, Nationwide Children’s Hospital, Center for Surgical Outcomes Research, Columbus, OH; † Department of Surgery, Nemours Surgical Outcomes Center, Nemours Children’s Health, Delaware Valley, Wilmington, DE.

**Keywords:** adolescent, laser epilation, pediatric, pilonidal, recurrence, treatment effects

## Abstract

**Objective:**

To investigate the heterogeneity of treatment effects (HTE) of laser epilation in preventing pilonidal disease recurrence through analysis of prespecified clinical factors.

**Background:**

Pilonidal disease is a common, painful disease affecting 1% of the population aged 15 to 30 years with postoperative recurrence rates as high as 30% to 40%.

**Methods:**

Single-institution randomized controlled trial from September 2017 to September 2022 with 1-year follow-up, including patients aged 11 to 21 years with pilonidal disease undergoing gluteal cleft laser epilation and standard care (improved hygiene and mechanical or chemical depilation) or standard care alone.

**Results:**

In total, 302 patients were enrolled with 151 randomized to each intervention. 1-year follow-up was available for 96 patients in the laser group and 134 in the standard care group. There were no significant differences in treatment effects based on sex, body mass index, previous disease, prior surgical excision, or annual household income (all *P* > 0.05). HTE was identified by race and ethnicity (*P* = 0.005) and health insurance type (*P* = 0.001). Recurrence among non-Hispanic white patients was 4% (3/75) with laser treatment and 31.6% (31/98) with standard care *versus* 38.9% (7/18) with laser treatment and 38.2% (13/34) with standard care among all other racial/ethnic groups. Recurrence rates among privately insured patients were 4.0% (3/75) with laser treatment and 33.3% (29/87) with standard care *versus* 36.8% (7/19) with laser treatment and 29.7% (11/37) with standard care in patients with public insurance.

**Conclusions:**

The effectiveness of laser epilation to reduce pilonidal disease recurrence rates may vary based on race and ethnicity and insurance type. Additional studies are warranted to investigate this potential HTE.

## INTRODUCTION

Pilonidal disease is a common and painful disease that affects approximately 1% of the population aged 15 to 30 years.^1–4^ Recurrence rates after operative intervention can be as high as 30% to 40%, emphasizing the need for preventative strategies to minimize recurrence.^5–17^ Previous studies have demonstrated an association between recurrence of pilonidal disease and prior episodes of disease, previous surgical excision, male sex, and higher body mass index (BMI).^7,18^ History of additional previous disease episodes has been reported to lead to higher recurrence rates while previous surgical excision has been reported to lead to lower recurrence rates.^7–10,18^ Male sex has been associated with higher overall disease incidence and higher recurrence rates.^19–25^ Obesity has also been associated with higher recurrence rates.^19,26–28^ Other variables that have been reported to be associated with increased recurrence rates include younger age, non-White race, midline surgical excision, lower income, lower education level, and being single or living alone.^7,10,18,29–31^

Recognizing the need for effective preventative strategies, this study reports planned analyses investigating the heterogeneity of treatment effects of laser epilation in preventing the recurrence of pilonidal disease for prespecified variables of interest, including number of prior episodes of disease, previous surgical excision, sex, and BMI in a single-institution randomized controlled trial that compared laser epilation and standard care to standard care along among adolescent and young adults with pilonidal disease.^32^ We hypothesized that the effectiveness of laser epilation on pilonidal disease recurrence would be less in patients who had more prior episodes of disease, had not had previous surgical excision, were male, and had higher BMI. Additional exploratory analyses based on self-reported race/ethnicity, household income, and insurance type were performed.

## METHODS

### Study Overview

This was a planned analysis of heterogeneity of treatment effects of laser epilation on pilonidal disease recurrence in a single-institution randomized controlled trial comparing laser epilation to standard of care in patients aged 11 to 21 years. This protocol is registered with ClinicalTrials.gov, and details of the design and methodology have previously been published (full protocol is available as Supplemental Material 1, http://links.lww.com/AOSO/A403), as have primary study results.^32,33^

Recruitment occurred between September 2017 and September 2022 and patients were followed for 1 year after randomization to undergo either laser epilation therapy with standard care or standard care alone. Patients were eligible for enrollment if they had a history of 1 or more episodes of pilonidal disease and no active disease at the time of enrollment. Patients were excluded if they had a history of photosensitivity or the presence of acute flare with active inflammation or infection at the time of enrollment. Participants were enrolled by a trained research coordinator and were randomly assigned in a 1:1 ratio using a randomized block scheme with block sizes of 4 or 6 with lengths chosen randomly with equal probability using a web-based system. Neither participants nor study team members were blinded to allocation, given the nature of the intervention.

Standard care included improved hygiene and mechanical or chemical depilation. Laser epilation entailed 1 treatment every 4 to 6 weeks for a total of 5 treatments. Depending on Fitzpatrick skin type as determined by the principal investigator, patients were treated with either a diode 810 nm (Fitzpatrick skin types I–IV) or Nd:YAG 1064 nm (Fitzpatrick skin types V–VI) laser device.^34^

Data were collected from patients and the electronic medical record and included demographic information, symptom and treatment history, and disease recurrence. Race and ethnicity data were collected to assess their associations with pilonidal disease recurrence. Participants self-identified with the following race and ethnicity categories: American Indian or Alaska Native, Asian, non-Hispanic Black, Hispanic, Native Hawaiian or Other Pacific Islander, multiracial, non-Hispanic White, or not reported. This study was approved by our institutional review board. Written informed consent and assent for participants less than 18 years of age were obtained from all participants. This study followed the Consolidated Standards of Reporting Trials (CONSORT http://links.lww.com/AOSO/A404) reporting guidelines.

### Primary Outcome

The primary outcome of this study was heterogeneity of treatment effects of laser epilation on pilonidal disease recurrence at 1 year. Heterogeneity of treatment effects exists when the effectiveness of laser epilation on recurrence was different in the treatment groups based on the level of a tested covariate (eg, surgical excision *vs*. no surgical excision). Heterogeneity of treatment effects was formally explored for prespecified variables of interest, including episodes of previous disease, previous surgical excision, sex, and BMI (kg/m^2^), and an exploratory analysis was conducted for additional factors, including race/ethnicity, health insurance type, and household income. Previous surgical excision was explored to test the hypothesis that patients with more severe disease (ie, they underwent surgery for it) may have a different response to laser epilation. The clinical presentation of recurrence with active pain, swelling, foul smell, wound drainage, and fevers, made it unlikely that a recurrence would be missed because these symptoms would prompt a call to the research team or presentation to medical attention. Recurrence was captured both through patients self-reporting symptoms to the research team as well as planned monthly follow-up by the research team through email, phone, or in clinic based on the patient’s preferred method for follow-up with additional verification by study staff through the medical chart. All instances of recurrence were confirmed by study staff based on the management of the patient’s acute concern. Since all patients were established with our designated pilonidal disease clinic, there was a high propensity for follow-up with our clinic.

### Sample Size and Power Calculations

Initial estimates for 1-year recurrence rates were expected to be 20% in the control group and 5% in the intervention group, based on available data. Due to concerns from the funding agency that recurrence rates may be lower than estimated because both groups were using the best available standard care (as compared to usual care), conservative estimates of 1-year recurrence of 12% in the control group and 2% in the intervention group were used for sample size calculations. Based on these estimates, under a group sequential design with 1 interim and 1 final analysis an overall 2-sided type I error rate of 5%, and power of 80%, the same size required was 123 participants per group. Assuming a 10% dropout rate over the course of the 1 year of follow-up, we planned to enroll 136 patients in each group for a total of 272 patients.

### Study Changes

Due to shutdowns during the COVID-19 pandemic, laser treatment appointments were temporarily halted, resulting in a group of 15 patients who were randomly assigned and enrolled in the laser epilation group but never started treatments and no longer wanted to participate when elective procedure suspensions were lifted. This unanticipated loss of patients from one arm necessitated increasing our sample size with the randomization of an additional 30 patients to maintain power.

### Statistical Analysis

Patients were described through summary statistics, using medians and interquartile ranges for continuous data and sums and proportions for categorical data. All outcome comparisons were conducted using an intention-to-treat approach. Treatment effect heterogeneity was explored by evaluating these factors as potential effect modifiers by including each in a model to include the main treatment effect, the main factor effect, and the interaction term for the treatment by factor.^35^ Treatment effects were estimated for each level of the factor and compared across groups. Identification of effect modification was made through tests of interaction in these models, which controlled the family-wise error rate of each of these comparisons at the 1% level (translating to a maximum family-wise error rate of 9%). Race/ethnicity was explored as 2 levels of non-Hispanic White *versus* all other racial/ethnic groups due to the small numbers of patients in the other categories. We performed *χ*^2^ and Fisher exact tests as appropriate to determine statistical significance between groups and differences in protocol adherence at 6 months among patients in the laser group. We considered a *P* value less than 0.05 significant. Statistical analysis was performed using SAS Enterprise Guide 8.1 (SAS Institute Inc., Cary, NC).

## RESULTS

**FIGURE 1.**
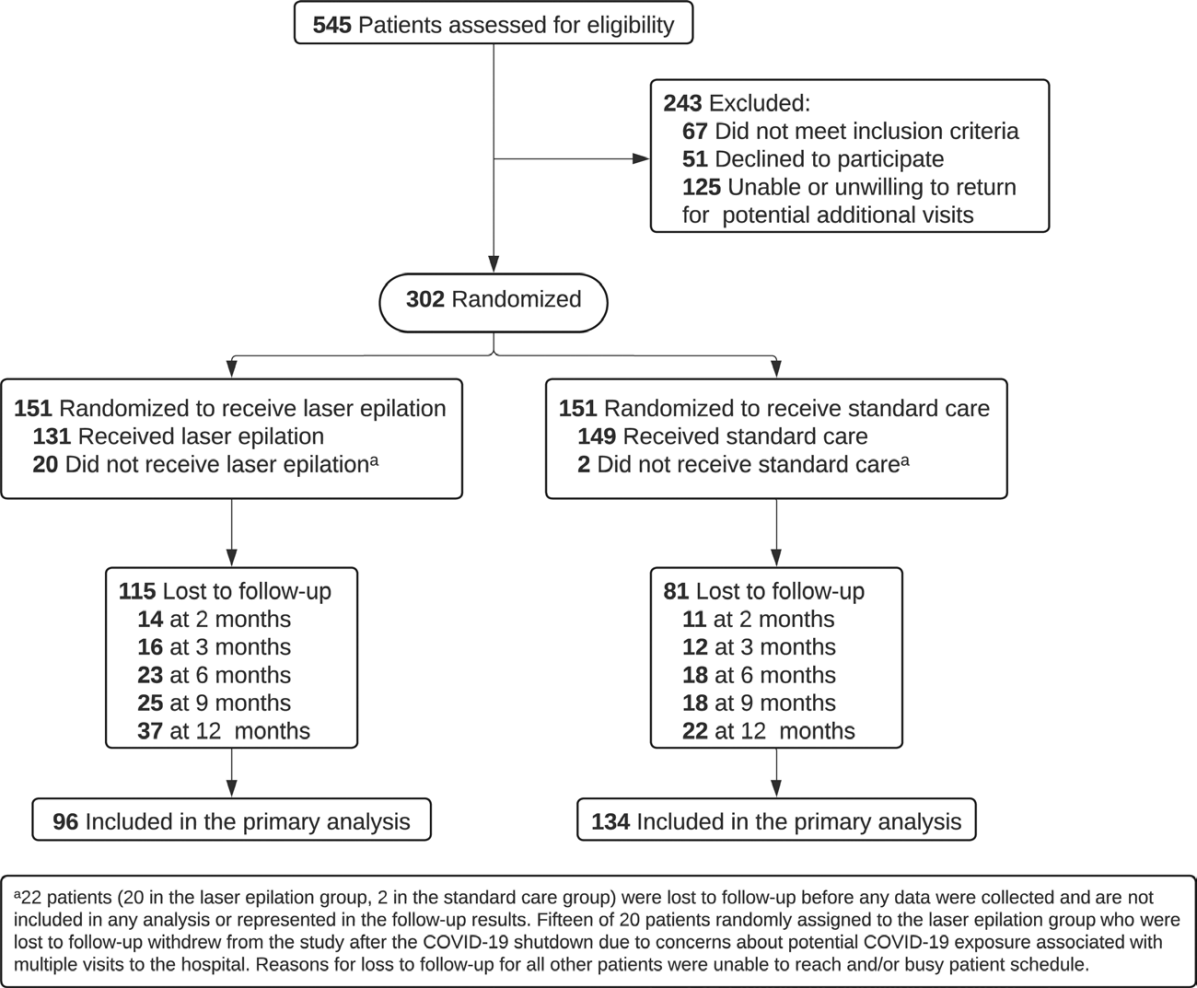
Study flow of standard care and laser epilation groups^32^ (with permission).

Three hundred and two patients were enrolled, with 151 randomized to laser epilation and 151 randomized to standard care (Fig. 1). Twenty patients in the laser group and 2 patients in the standard care group were lost to follow-up before any data were collected so they were excluded from all analyses. One-year follow-up based on electronic medical record review was available for 96 patients in the laser group and 134 in the standard care group.

Overall, the median age for both groups was 17 years (Table 1) and non-Hispanic White race/ethnicity was most common (75.0%, 210/280). Forty percent (112/280) of patients were obese. Most patients (73.6%, 206/280) had only 1 prior episode of disease before trial enrollment, and 16.8% (47/280) had required prior surgical excision. Private health insurance was most common (67.1%, 188/280) and annual household income most commonly exceeded $100,000 (43.6%, 122/280).

**TABLE 1.** Baseline Patient Demographics and Clinical Characteristics

	Overall n (%)	Laser n (%)	Standard care n (%)
Total number of patients^*^	280	131 (46.8)	149 (53.2)
Age, years, median (IQR)	17 (15, 18)	17 (15, 18)	17 (15, 18)
Male sex	157 (56.1)	71 (54.2)	86 (57.7)
Race/ethnicity			
Non-Hispanic White	210 (75.0)	103 (78.6)	107 (71.8)
Non-Hispanic Black	31 (11.1)	8 (6.1)	23 (15.4)
Hispanic	6 (2.1)	3 (2.3)	3 (2.0)
American Indian/Alaska Native	2 (0.7)	1 (0.8)	1 (0.7)
Asian	8 (2.9)	3 (2.3)	5 (3.4)
Native Hawaiian or Other Pacific Islander	2 (0.7)	2 (1.5)	0 (0)
Multiracial	15 (5.4)	8 (6.1)	7 (4.7)
Not reported	6 (2.1)	3 (2.3)	3 (2.0)
Body mass index, kg/m^2^			
Underweight or normal weight (<25)	93 (33.2)	49 (37.4)	44 (29.5)
Overweight (25–30)	63 (22.5)	25 (19.1)	38 (25.5)
Obese (>30)	112 (40.0)	52 (39.7)	60 (40.3)
Not documented	12 (4.3)	5 (3.8)	7 (4.7)
Prior episodes of disease			
1	206 (73.6)	93 (71.0)	113 (75.8)
2	57 (20.4)	28 (21.4)	29 (19.5)
≥3	17 (6.1)	10 (7.6)	7 (4.7)
Prior surgical excision	47 (16.8)	24 (18.3)	23 (15.4)
Type of health insurance			
Public	78 (27.9)	32 (24.4)	46 (30.9)
Private	188 (67.1)	95 (72.5)	93 (62.4)
Other	4 (1.4)	1 (0.8)	3 (2.0)
None	10 (3.6)	3 (2.3)	7 (4.7)
Annual household income			
Less than $25,000	22 (7.9)	9 (6.9)	13 (8.7)
$25,000–$49,999	55 (19.6)	22 (16.8)	33 (22.2)
$50,000–$99,999	73 (26.1)	36 (27.5)	37 (24.8)
$100,000 or more	122 (43.6)	62 (47.3)	60 (40.3)
Not reported	8 (2.9)	2 (1.5)	6 (4.0)

* 20 patients in the laser group and 2 patients in the standard care group were lost to follow-up before any data were collected so were excluded from all analyses.

There was no evidence of treatment effect heterogeneity by previous episodes of disease, previous surgical excision, sex, or BMI (Table 2; all *P* > 0.05). The effect of laser treatment on pilonidal disease recurrence differed by patient race/ethnicity (*P* = 0.005) and type of health insurance (*P* = 0.001). Recurrence among non-Hispanic White patients was 4% (4/75) with laser treatment and 31.6% (31/98) with standard care alone *versus* 38.9% (7/18) with laser treatment and 38.2% (13/34) standard care alone among all other racial/ethnic groups. Privately insured patients had recurrence rates of 4% (3/75) with laser treatment and 33.3% (29/87) with standard care alone versus 36.8% (7/19) with laser treatment and 29.7% (11/37) with standard care alone in patients with public insurance. Likelihood of patients in the laser treatment arm undergoing all 5 laser treatments within 6 months did not differ based on race/ethnicity or type of health insurance (Table 3).

**TABLE 2.** Heterogeneity in the Effect of Laser Therapy on Recurrence of Pilonidal Disease Among Adolescents and Young Adults

	Laser		Standard Care		
Variable of Interest	Total n	Recurrence n (%)	Total n	Recurrence n (%)	*P* Value*
Planned analysis					
Sex					0.33
Female	45	7 (15.6)	59	22 (37.3)	
Male	51	3 (5.9)	75	23 (30.7)	
Body mass index^†^, kg/m^2^					0.24
Overweight or obese	52	8 (15.4)	89	31 (34.8)	
Underweight or normal weight	39	2 (5.1)	41	13 (31.7)	
Prior episodes of disease					0.35
1	69	5 (7.3)	99	32 (32.3)	
2	20	4 (20.0)	28	9 (32.1)	
≥3	7	1 (14.3)	7	4 (57.1)	
Prior surgical excision					0.60
Yes	16	1 (6.3)	21	7 (33.3)	
No	80	9 (11.3)	113	38 (33.6)	
Exploratory analysis					
Race/ethnicity^†^					0.005
Non-Hispanic White	75	3 (4.0)	98	31 (31.6)	
All other racial/ethnic groups	18	7 (38.9)	34	13 (38.2)	
Type of health insurance					0.001
Public	19	7 (36.8)	37	11 (29.7)	
Private	75	3 (4.0)	87	29 (33.3)	
Annual household income^†^					0.09
Less than $25,000	8	2 (25.0)	11	5 (45.5)	
$25,000–$49,999	11	5 (45.5)	27	12 (44.4)	
$50,000–$99,999	27	1 (3.7)	35	13 (37.1)	
$100,000 or more	50	2 (4.0)	56	15 (26.8)	

* *P* values are for the interaction term from logistic regression models that were fit in the entire cohort and that included treatment group, the effect modifier of interest, and an interaction between these.

† Patients with unreported values were excluded from analysis.

**TABLE 3.** Proportion of Patients in the Laser Care Group Who Underwent all 5 Laser Treatments Within 6 Months

	Total n	Underwent all Laser Treatment Sessions in 6 Months n (%)	*P* Value*
Race/ethnicity^†^			0.70
Non-Hispanic White	103	62 (60.2)	
All other racial/ethnic groups	25	14 (56.0)	
Type of health insurance^†^			0.19
Public	32	16 (50.0)	
Private	95	60 (63.2)	

* *P* values compare the proportion of non-Hispanic Whites to all other racial/ethnic groups and public insurance to private insurance among patients who underwent all 5 laser treatments within 6 months.

† Patients with unreported values were excluded from analysis.

## DISCUSSION

The effectiveness of laser epilation on pilonidal disease recurrence did not differ based on previous episodes of disease, previous surgical excision, sex, or BMI. In exploratory analyses, laser epilation was associated with a greater reduction in recurrence rates among patients of non-Hispanic White race/ethnicity compared with all other patients and among privately insured compared with publicly insured patients. Further investigation into these differences in the treatment effects of laser epilation is warranted to try to promote equitable health care outcomes across all patients with pilonidal disease.

Contrary to expectations, our study did not reveal significant differences in treatment effects based on sex, BMI, the number of episodes of previous disease, or history of surgical excision. This is in contrast to prior studies that demonstrate higher rates of recurrence based on these factors.^7,9,10,18,29–31^ While our patient population was predominantly male and many patients were overweight or obese, laser epilation did not have differences in effectiveness based on sex or BMI. Components designed to address hygiene and interval hair growth were included as part of standard care in this study and may account for our differing results; the description of what was included as standard care in previous studies was incomplete and did not allow us to determine if there were systematic differences in what was recommended between studies. Additionally, the lack of difference in treatment effect seen based on previous disease or history of surgical excision may be due to the small proportion of patients in our study cohort who had more than 2 episodes of disease or who required previous surgical excision. Our institution has a designated clinic for the management of pilonidal disease with patient referral earlier in their disease course, which likely accounts for the lower number of prior episodes of disease and prior surgical excision among our patient population.^36^

A significant difference in the effectiveness of laser epilation to decrease recurrence rates was identified among non-Hispanic White patients compared with other racial/ethnic groups. This finding of potential racial/ethnic disparities in treatment response should be interpreted with caution as it was an exploratory analysis and many racial/ethnic categories had small numbers of patients in our study. Additionally, the decreased effectiveness of laser epilation based on race/ethnicity may be a surrogate for differences in treatment effectiveness based on skin color. Previous retrospective observational research by Salimi-Jazi, et al^29^ demonstrated a smaller reduction in pilonidal disease recurrence after laser epilation in darker skin adolescent patients (Fitzpatrick skin types V–VI) compared with those of lighter skin (Fitzpatrick skin types I–IV) after controlling for other confounding factors.^29,34^ Our study attempted to minimize potential issues related to laser epilation treatment tolerance based on skin color as a cause for differences in treatment effects. Patients in the laser epilation group were treated with a different laser based on skin color with patients with lighter skin color (Fitzpatrick skin types I–IV) being treated with a Diode 810 nm laser and those with darker skin color (Fitzpatrick skin types V–VI) with an Nd:YAG 1064 nm laser.^37–43^ Both lasers are FDA-approved for hair removal in all areas of the human body, including the back and perineum. Prior studies demonstrated successful removal of abnormal hair growth in patients with darker skin pigmentation with treatment with the Nd:YAG laser without serious adverse side effects.^37–40,44,45^ In contrast, shorter wavelength lasers, such as the Diode 810 nm, have been found to cause blistering and discoloration in patients with higher Fitzpatrick skin types, making Diode use less favorable and Nd:YAG use preferable for these patients.^46–48^ Further investigation is warranted with multicenter trials that include targeted numbers of patients with specific demographics and skin types to investigate differences in treatment effects based on skin color and race/ethnicity.

Our study identified a substantial impact of health insurance type on recurrence rates, with laser treatment demonstrating higher efficacy in privately insured patients. The potential impact of insurance status on a clinical presentation for evaluation of concerns for recurrence should have been attenuated through patients having multiple no-cost avenues for assessment for recurrence during the study, including planned monthly follow-up by the research team through email, phone, or in clinic based on the patient’s preferred method for follow-up. Ultimately, the relationship we observed may be mediated by better access to care and healthcare outcomes associated with higher socioeconomic status.^49–51^ It is possible that health insurance status is also associated with other social determinants of health, such as access to transportation, and health literacy, with patients with public insurance being more likely to have negative social determinants of health and lower health literacy, which may have led to worse outcomes. Regardless, differences in outcomes based on health insurance type demonstrate the need for equitable access to advanced treatments and raise important considerations for emphasizing the need for interventions to address these differences.

Overall, the likelihood of patients in the laser arm adhering to the treatment protocol within the study timeframe did not differ significantly based on race/ethnicity and type of health insurance. This suggests that factors influencing treatment adherence, such as discomfort related to laser epilation, scheduling conflicts, and available transportation, may not account for the observed differences in treatment outcomes, even in the context of the COVID-19 pandemic. However, it is essential to further explore potential barriers to treatment adherence, as this may have implications for the overall effectiveness of laser epilation in preventing recurrence.

### Limitations

There are a few key limitations to consider. First, because this was a single-institution study, generalizability to other institutions using different methods for pilonidal disease management or significant intra-institutional variability in disease management may be limited. This is true both for referral patterns and management following surgical referral. All clinicians that staff the pilonidal disease clinic at our institution follow the same algorithm for the management of this disease process, which includes strategies for conservative treatment and timing of escalation to surgical intervention.^36^ Future research is warranted to include multicenter trials with extended follow-up. Second, our exploratory analysis of additional factors that may contribute to the heterogeneity of treatment effects, including race/ethnicity and insurance type are limited by small sample sizes for several categories. Furthermore, the heterogeneity of treatment effects detected based on race/ethnicity may be due to differences in skin color. Future adequately powered trials are needed to investigate these potentially important differences in treatment effects based on skin color and race/ethnicity. Third, longer-term follow-up may have identified delayed episodes of recurrence occurring more than a year after treatment. Finally, due to the loss to follow-up, there was missing data for primary and secondary outcomes. This was accounted for in our analysis via additional sensitivity analyses and censoring from longitudinal analyses, but there was potential for misclassification due to incomplete treatment adherence, which may have been exacerbated by the COVID-19 pandemic.

## CONCLUSIONS

Our study provides valuable insight into the heterogeneity of laser epilation treatment effects for preventing pilonidal disease recurrence. Exploratory analyses suggest potential differences based on race/ethnicity and health insurance type that necessitate further investigation. Understanding the complex interplay of these factors is crucial for achieving equitable healthcare outcomes across diverse patient populations. Planned research efforts will explore these potential differences in treatment effects of laser epilation based on race/ethnicity and skin color in multicenter clinical trials.

## AUTHORS CONTRIBUTION

Conceptualization: J.C., K.J.D., and P.C.M.; data curation: C.L. and L.G.; formal analysis: J.C. and L.A.; data visualization: K.C.B., C.L., and L.G.; supervision: C.C., K.J.D., and P.C.M.; original draft: K.B.; critical review and revision: K.B., C.L., J.C., L.A., L.G., C.C., K.J.D., and P.C.M.

## Supplementary Material


