# Supplementary material for: Heterogeneity of Treatment Effects of Laser Epilation on Pilonidal Disease Recurrence: A Randomized Clinical Trial
Source: Ann Surg Open. 2024 Sep 3;5(3):e488. doi: 10.1097/AS9.0000000000000488 (PMC11415133; doi:10.1097/AS9.0000000000000488)
Supplement: Supplementary file 1 [file as9-5-e488-s001.pdf]

PROTOCOL TITLE:

A randomized controlled trial of laser hair depilation in adolescents with pilonidal disease

PRINCIPAL INVESTIGATOR:

Name: Peter Minneci, MD, MHSc.
Department/Center: Division of Pediatric Surgery/ Center for Surgical Outcomes Research
Telephone Number: (614) 722-5922
Email Address:Peter.Minneci@nationwidechildrens.org

VERSION NUMBER/DATE:

v.10 – 2/15/2021

REVISION HISTORY

Revision #	Version Date	Summary of Changes	Consent Change?
5	11/1/2019	Added contact with PCP at 12 months	Yes
6	11/19/2019	Increased 12 month research debit card to $50	Yes
7	1/7/2020	Added marketing campaign	No
8	4/28/2020	COVID adjustments	No
9	6/9/2020	Added electronic consent	No
10	2/15/2021	Increase sample size from 272 to 302	Yes

1.0 Study Summary

**Study Title**	A randomized controlled trial of laser hair depilation in adolescents with pilonidal disease
**Study Design**	Randomized Controlled Trial
**Primary Objective**	The present study is a follow-up study to IRB16-00104. The objective of this study is to perform a randomized controlled trial of laser hair depilation + chemical/mechanical depilation versus chemical/mechanical depilation only to examine outcomes related to recurrence of pilonidal disease. Recurrence is defined as a new development of pilonidal abscess, folliculitis, or draining sinus after treatment which would require antibiotic treatment, additional surgical incision and drainage or excision.
**Secondary Objective(s)**	The secondary objective is to compare adverse outcomes, treatment-related pain, length of stay, disability days, costs and burden of care and quality of life measures, and disease-related stigma and attitudes between those that receive laser hair depilation plus mechanical/chemical hair depilation with those that receive only mechanical/chemical hair depilation.
**Research Intervention(s)/ Investigational Agent(s)**	Laser hair depilation: It was determined by the Director of the Drug and Device Development Services and the Institutional Review Board (IRB) at NCH that an investigational device exemption is not necessary because we are using an approved device for investigational purposes with non-significant risk. Laser use in this study poses minimal risk to the patient and these minimal risks are similar to the risks associated with hair removal in other regions of the body for which these devices are already FDA approved.
**IND/IDE #**	N/A
**Study Population**	Any patient age 11-21 years old with history of at least one episode of pilonidal disease and who does not currently have an actively inflamed pilonidal sinus/ history of photosensitivity.
**Sample Size**	302
**Study Duration for individual participants**	Each patient will be involved in the study for two years. Randomized and consented patients will either visit the surgery clinic for 1 treatment every 4-6 weeks to obtain a total of 5 treatments if randomized to laser treatment or have an initial clinic visit with subsequent monthly assessments by email or phone for the first five months. We will continue to follow-up with laser participants at 6, 9, 12, 18 and 24 months from the initial treatment. Participants in the standard care arm will have a follow-up for the 6, 9, and 12 month time points. At the 12 month time point, patients randomized

	to the standard care group will be offered laser treatment. If these patients choose to receive the elective laser treatments, they will be brought back into the clinic and re-consented by the study staff prior to their first treatment. They will have the option to commit to one or two additional years in the study and will be followed in the same manner as the patients initially randomized to the laser intervention group. Data will be collected from the patients per the same follow-up surveys at 1, 2, 3, and 4 months and post-treatment follow-up surveys at 6, 9, and 12 months. Control arm participants who do not receive the elective laser treatments at the 12 month time point will continue to follow up at 18, and 24 months from the initial treatment.<br><br>Beginning March 16, 2020 the Abigail Wexner Research Institute halted all non-essential research study appointments in response to the COVID-19 pandemic. Laser arm patients whose treatments are delayed will have a gap between treatments longer than 4-6 weeks. This gap will vary between the 1, 2, 3, and 4 month follow-ups and some may occur during the patients 6 month, 9 month or 12 month follow-ups.<br><br>The gap in the laser treatments caused by COVID-19 also created a gap in when we could begin laser treatments in newly enrolled patients by 1-3 months. Since a laser patient's participation in the study begins at their first laser treatment, no data has been collected on these patients. Patients in the group have reported no longer being interested/able to receive treatments due to concerns about COVID and visits to the hospital to receive laser treatments. This group of about 15 patients was not anticipated for our sample size calculation but are needed for the primary outcomes data. In order to maintain the required power in our analyses, our statistician recommends enrolling and randomizing an additional 30 patients, thereby increasing our sample size to 302 patients (151 in each group).
**Study Specific Abbreviations/ Definitions**	

2.0 Objectives

2.1 The present study is a follow-up study to IRB16-00104. The objective of this study is to perform a randomized controlled trial of laser hair depilation + chemical/mechanical depilation versus

chemical/mechanical depilation only to examine outcomes related to recurrence of pilonidal disease. Recurrence is defined as a new development of pilonidal abscess, folliculitis, or draining sinus after treatment which would require antibiotic treatment, additional surgical incision and drainage or excision.

The secondary objective is to compare adverse outcomes, treatment-related pain, length of stay, disability days, costs and burden of care and quality of life measures, and disease-related stigma and attitudes between those that receive laser hair depilation plus mechanical/chemical hair depilation with those that receive only mechanical/chemical hair depilation.

2.2 We recently completed a single arm pilot study at NCH which demonstrated the safety and tolerance of laser depilation in adolescents and young adults (IRB16-00104). We enrolled 13 patients and treated them with either an 810 nm wavelength laser (for Fitzpatrick skin types I-IV) or Nd:YAG laser (for Fitzpatrick skin types V-VI) monthly for 5 treatments. Twelve patients completed at least 4 laser depilation treatment sessions with 100% tolerability and no second degree burns. No patients were unable to complete a treatment session due to discomfort. All 13 patients remain recurrence-free at 13 months after the initiation of treatment with significantly diminished hair growth noted in all patients after 3 treatments. These results confirm that laser hair depilation is both safe and well tolerated in in adolescents and young adults with pilonidal disease and may represent a promising therapy to decrease disease recurrence.

3.0 Background

3.1 Pilonidal disease is a common problem among adolescents and young adults. It affects 26 per 100,000 people with an incidence of 1.1%.[1,5] It is characterized by the development of epithelialized tracks and sinuses within the natal cleft extending up to the level of the coccyx posteriorly (the area between the buttocks and posterior to the anus). Entrapped hair follicles become infected resulting in acute and chronic wounds and draining sinuses that can incur both short term and long term morbidity, disability, and poor quality of life.[2] The most often seen initial presentation is that of a painful mass in the sacrococcygeal region.[7] Initial treatment is usually with antibiotics and/or surgical incision and drainage of the inflamed and often infected cystic cavity. Recurrence rates have been conservatively reported at 16% and higher than 30% in some patient populations.[2,3]

The morbidity of this disease is very significant. Patients, usually in their adolescence and young adulthood, may endure chronic pain/inflammation, drainage of sinus tracts associated with significant odor, and occasional negative-pressure wound VAC dressings which are cumbersome. When disease is active, these patients must lay prone at all times when

attempting to sleep and are unable to sit due to their wounds; patients with recurrent disease report losing months to years of school and work obligations related to the treatment and chronic nature of their wounds as well as the emotional toll related to an embarrassing problem in a sensitive body area. This disease remains a source of frustration for patients and their families; they desire a durable treatment.

For patients with recurrent disease, there are both medical and surgical methods to treat and/or palliate pilonidal disease. Each of these methods incurs a different risk-benefit profile and has been associated with significant morbidity. Overall, surgical methods appear to be more durable with long term success rates ranging from 84 to 89% However, surgical excision and reconstruction of this area can be associated with significant morbidity and cost. Recurrence rates of pilonidal disease after resection have been reported to be 11%.[2,3] Furthermore, wound issues after resection with primary closure have been reported to be as high as 30%.[4] Medical therapy is largely dependent upon continued lifelong hair removal either from shaving or chemical depilation, meticulous hygiene to the area, and recurrent courses of antibiotics with intermittent need for both office-based, and operating room incision and drainage procedures. This approach to pilonidal disease incurs a chronicity that can lead to longer term disability and reduced quality of life. In fact, the presence of pilonidal disease and its complications is an accepted indication for Disability Insurance qualification.6 It has been noted by several authors that an optimal treatment for pilonidal disease has yet to be identified.[2,3]

A historical chart review (IRB 16-00648) of patients with pilonidal disease treated at Nationwide Children's Hospital (NCH) demonstrated an overall recurrence rate of 36% with recurrence rates of 32% after initial treatment with antibiotics, 36% after an incision and drainage procedure, and 35% after surgical excision. The 1 year recurrence rate is 20%; therefore, the majority of patients that experience a recurrence will do so in the first year. These high rates necessitate identifying treatments that can effectively decrease the recurrence of pilonidal disease.

A small number of studies indicate that laser hair depilation of the natal cleft appears to be protective for pilonidal disease recurrence.[4] Due to these limited data, the mechanism of this disease, and the absence of randomized controlled data in the alternative treatment modalities of pilonidal disease, we believe that laser depilation should be studied as a first line therapy. We recently completed a pilot study at NCH (IRB16-00104) which demonstrated the safety and tolerance of laser depilation in adolescents and young adults. The goal of this study is to determine the efficacy of this treatment in a single center, randomized controlled trial, comparing laser depilation to standard of care in pediatric patients with pilonidal disease.

We recently completed a single arm pilot study at NCH which demonstrated the safety and tolerance of laser depilation in adolescents and young adults (IRB16-00104). We enrolled 13 patients and treated them with either an 810 nm wavelength laser (for Fitzpatrick skin types I-IV) or Nd:YAG laser (for Fitzpatrick skin types V-VI) at 4-6 week intervals for 5 treatments. Twelve patients completed at least 4 laser depilation treatment sessions with 100% tolerability and no second degree burns. No patients were unable to complete a treatment session due to discomfort. All 13 patients remain recurrence-free at 13 months after the initiation of treatment with significantly diminished hair growth noted in all patients after 3 treatments. These results suggest that laser hair depilation is both safe and well tolerated in in adolescents and young adults with pilonidal disease and may represent a promising therapy to decrease disease recurrence.

3.2 Surgical Management of Pilonidal Disease: Studies related to the surgical management of pilonidal disease have largely focused on the technical aspects of resection and reconstruction. Over one hundred studies in the literature have reported various methods for managing this disease, but with inconsistent methodology and consistently unsatisfactory outcomes. The ideal operation for pilonidal disease should eradicate the disease, minimize recurrence, and carry low morbidity and disability. Incision and drainage has been the primary treatment in cases of acute presentation. After incision and drainage, overall successful healing has been reported to be approximately 60%, whereas the remaining patients required an additional excision procedure before closure of these wounds. For patients with recurrent disease, wide local excision with primary or secondary closure, with consideration of off-midline closure, has been widely accepted. However, wide local excision in this body region is associated with significant morbidity including a post-operative wound complication rate of 30%. Marsupialization for pilonidal disease entails incising the sinus tracts without excision of normal tissue resulting in smaller wounds, limited tissue trauma, and shorter recovery.[8] However, reported duration of healing and durability has been variable.

Medical Management of Pilonidal Disease: Standard medical management of pilonidal disease utilizes hair depilation and local wound care with intermittent antibiotic administration for flares.

Phenol solutions have also been tried in an attempt to sclerose sinus tracts. This involves one or more injections into the affected chronic sinus tracts until filled, removal of sinus hairs and debris with forceps, as well as local shaving. Smaller prospective series have demonstrated success rates ranging from 60% to 95%.[9-12] In cases of recurrent chronic sinus disease, phenol injection and local depilatory cream application on a weekly basis have shown low subsequent recurrence rates (0%–11%) at extended follow-up.[13] In chemical depilatory creams, common active ingredients are calcium thioglycolate or potassium thioglycolate, which breaks down the

disulfide bonds in keratin and weakens the hair so that it is easily scraped off where it emerges from the hair follicle. The hair follicle does remain with hair regrowth noted in 2 to 5 days and there can be some local skin irritation on the site of application. Some studies have explored the utility of certain skin glues to close the chronic tracts associated with chronic pilonidal disease. One such study utilized Fibrin glue been injected into these chronic tracts after simple curettage with some positive effect.[14] Overall, studies of sclerosing agents and glue have yielded variable results with limited long term efficacy.

Laser Depilation in Pilonidal Disease: In several retrospective studies and small prospective studies, laser hair removal has shown promise as an adjunct therapy to decrease recurrent infections and decrease the need for repeat surgery in adults and older adolescents. Lukish et al. published a retrospective review of twenty-eight teenage patients with pilonidal disease who underwent initial surgical excision of their presenting abscess. Laser depilation was found to be well tolerated with only one patient presenting with recurrent disease after a mean follow up of 24 months.[4] Landa et al. determined that their series of 6 patients with recurrent pilonidal disease saw progressive resolution of folliculitis after 3 to 11 treatments with no need for recurrent excision. No complications or recurrence were reported.[17] Schulze et al reported their experience of eighteen men and five women treated with laser depilation from 2001 to 2004. All patients had experienced recurrent folliculitis and had undergone some form of drainage procedure or prior excision. After surgical excision of the affected area, a Vasculite Plus laser was used for the depilation treatments. Each session involved 9 to 12 treatments and the patients underwent an average of two sessions. All 19 of the patients that remain in follow-up report no recurrence of their folliculitis or need for further surgical procedures.[16] These studies show a positive effect with laser depilation in combating recurrent disease. However, there remains a paucity of prospective controlled studies related to this treatment modality with minimal data reported on the effectiveness of laser depilation in children with pilonidal disease. Prospective studies of laser hair removal in pediatric patients to assess safety and generate estimates of effectiveness is warranted.[15-17]

4.0 Study Endpoints

4.1 The primary outcome is the rate of recurrent pilonidal disease at 1 year defined as development of a new pilonidal abscess, folliculitis, or draining sinus after treatment, which would require antibiotic treatment, additional surgical incision and drainage, or excision within 1 year of enrollment.

Secondary outcome include: differences in disability days of the patient at 1 year, disability days of the caregiver at 1 year, HRQOL, health care satisfaction, disease-related attitudes and perceived stigma, rates of

pilonidal disease-related complications, rates of pilonidal disease related procedures (incision and drainage), surgical excision, and post-operative complications, and rates of compliance with recommended treatment.

A summary of the outcomes that will be assessed are listed in the table below (Table 2).

Table 2: Outcomes to be assessed

Outcome	Initial visit	1 month	2 month	3 month	4 month	6 month	9 month	12 month	18 month	24 month
Pilonidal disease recurrence (Primary Outcomes)	X	X	X	X	X	X	X	X	X	X
Disability days of the patient	X	X	X	X	X	X	X	X	X	X
Disability days of the caregiver	X	X	X	X	X	X	X	X	X	X
Compliance with recommended treatment	X	X	X	X	X	X	X	X	X	X
Pilonidal disease related complications	X	X	X	X	X	X	X	X	X	X
Emergency Department visits	X	X	X	X	X	X	X	X	X	X
Readmissions	X	X	X	X	X	X	X	X	X	X
Additional surgical or interventional procedures	X	X	X	X	X	X	X	X	X	X
Post-operative complications	X	X	X	X	X	X	X	X	X	X
Healthcare-associated costs	X	X	X	X	X	X	X	X	X	X
Incremental cost-effectiveness								X		
Satisfaction with Health Care		X			X		X			
Health Related Quality of Life	X			X		X		X		
Disease-related stigma	X			X			X			
Disease-related attitudes	X		X			X		X		

4.2 Data will be monitored by research team members once a week to ensure that all data (clinical data and questionnaires) collected are correctly completed. The proportion of patients that have a study related SAE (second degree burn or pain score >8) will be calculated after every group of 10 patients have completed their course of treatment. If the lower limit of the 95% confidence interval for this proportion exceeds 10%, the trial will be stopped. A Data Safety and Monitoring Committee (DSMC) has been formed and will meet every 6 months throughout the period during which patients are being recruited and through their first year of follow-up. The DSMC will review data provided by the primary study statistician and other study staff involved in data management and analysis. Dr. Adam Goldin (pediatric surgeon, Seattle Children's Hospital) will act as the chairman of the DSMC, and will serve alongside Dr. Martin Blakely (pediatric surgeon, Vanderbilt) and Dr. Joseph Rausch (Statistician/Researcher, Nationwide Children's Hospital). The study principal investigator will be made aware of all adverse events as they occur and a quarterly review of all adverse events (AEs) that occur in the trial will be performed by the study team. All unexpected non-serious AEs and SAEs relating to participation in the study will be reported verbally and in writing to the study PI and NCH IRB. The verbal report will occur within 48 hours of the occurrence. The written report of a SAE (e.g., death or life-threatening adverse event) will be reported within 7 days.

Table 3. Number of patients with an SAE needed to stop the study early:

Number of laser treatment patients	If this many or more with an SAE, the study will be stopped	95% LCL if this number with an SAE	95% UCL if this number with an SAE
10	3	0.103	0.608
20	5	0.108	0.473
30	7	0.115	0.412
40	8	0.102	0.350
50	10	0.111	0.332
60	11	0.104	0.301
70	13	0.111	0.294

An interim analysis is planned when a quarter of the planned total patients (26 patients in each group) have completed their 1 year follow-up and a final analysis will be performed when all patients have completed 1 year of follow up. These two analyses have been unequally spaced in order to ensure that the interim analysis is performed before study enrollment is complete. The interim analyses will be interested in assessing futility of the primary endpoint; the trial will be monitored based on the primary outcome of the proportion of patients with recurrent pilonidal disease and will employ the error spending function approach described by Lan and Demets and the error spending function described by O'Brien and Fleming.

Throughout the trial, the rate of pilonidal disease recurrence at 6 months and 1 year and the tolerance of laser treatments will be monitored for safety. Tolerance to the laser treatments will be monitored by examining for study related serious adverse events (SAE) which include severe pain (defined as a maximum pain score of 8 or above during or within 24 hours of treatment) or second degree burns. The rates of SAEs will be calculated after every group of 10 patients has completed the course of treatment. The percentage of patients with either a second degree burn or a maximum pain score of 8 or above during or after treatment will be estimated. If the lower 95% confidence limit of this proportion exceeds 10%, the trial will be stopped (see Table 3 in the Protection of Human Subjects section for the stopping threshold at each interim safety analysis). The 10% level was chosen based on input from patients involved in our feasibility and tolerability pilot in which they expressed that even if they had experienced a high pain score or minor burn, they would like to continue receiving therapy as these both are self-limited events with minimal long term morbidity. These rates will be regularly reported to the Data and Safety Monitoring Committee (DSMC).

To verify the incident of a suspected recurrence during the course of the study, staff may request that the patient consent to release their protected health information from external entities. This would require the patient or the patient's personal representative to sign an MR-9 Authorization form.

Any adverse events such as an inability to tolerate the laser therapy or a recurrence will be tracked. Since recurrence is an expected adverse event it will not be reported; however, it will be monitored and if we detect an increased rate of recurrence with laser therapy, this will be reported and the trial will be stopped. Mild to moderate pain (pain score 7 or lower) secondary to laser therapy is also consider an expected adverse event and will not be reported. SAEs due to laser treatment include severe pain (pain score 8 or higher) and deep second degree burns.

It is also possible that AE might occur that are not directly related to the study. As such, all AE will be classified as not related (clearly unrelated to study participation), possibly related (temporally related to study participation but could have been caused by other factors), or probably related (temporally related to study participation and cannot be reasonably explained by other factors) to study participation. The clinical study team will review all AE as they occur and determine the seriousness and relatedness of them.

We do not expect any severe AE in either study group. The clinical study team will review any SAE as they occur. All SAEs deemed probably related to the study (and all deaths) will be reported to the IRB within 72 hours of discovery.

5.0 Study Intervention/Investigational Agent

5.1 Subjects randomized to the laser depilation arm will visit the surgery clinic for 1 treatment every 4-6 weeks to obtain a total of 5 treatments. Fitzpatrick skin type classification will be assessed during the initial visit. The Fitzpatrick skin type classification is based on the level of pigmentation of the skin and its response to ultraviolet light (See Appendix). It will be used to select the best laser to perform hair removal for each patient. The laser treatment group will consist of an 810 nm (for Fitzpatrick skin types I-IV) or Nd:YAG (for Fitzpatrick skin types V-VI) 28 joule application at auto pulse duration for 400 ms. A cooling platform and application of 7% lidocaine/ 7% tetracaine cream, applied 45 minutes prior to treatment, will minimize any discomfort associated with the heat of the laser treatments.

The two lasers that will be used in this study (810 nm or Nd:YAG) are both FDA approved for hair removal in all areas of the human body including the back and perineum. Pilonidal disease is not an FDA approved indication for laser hair removal; however, it is being performed commonly in clinical practice. It was determined, by the Director of the Drug and Device Development Services and the Institutional Review

Board (IRB) at NCH that an investigational device exemption is not necessary because we are using an approved device for investigational purposes with non-significant risk. Laser use in this study poses minimal risk to the patient and these minimal risks are similar to the risks associated with hair removal in other regions of the body for which these devices are already FDA approved. Furthermore, our institutional pilot study confirmed the safety and tolerability of these specific laser machines in an adolescent and young adult population.

5.2 Drug/Device Handling: If the research involves drugs or device, describe your plans to store, handle, and administer those drugs or devices so that they will be used only on subjects and be used only by authorized investigators. N/A – We are using a approved device for investigational purposes with non-significant risk.

- If the control of the drugs or devices used in this protocol will be accomplished by following an established, approved organizational SOP (e.g., Research Pharmacy SOP for the Control of Investigational Drugs, etc.), please reference that SOP in this section. – N/A

5.3 If the drug is investigational (has an IND) or the device has an IDE or a claim of abbreviated IDE (non-significant risk device), include the following information: N/A

- Identify the holder of the IND/IDE/Abbreviated IDE. N/A
- Explain procedures followed to comply with sponsor requirements for FDA regulated research for the following: N/A

	Applicable to:		
FDA Regulation	***IND Studies***	***IDE studies***	***Abbreviated IDE studies***
21 CFR 11	***X***	***X***	
21 CFR 54	***X***	***X***	
21 CFR 210	***X***		
21 CFR 211	***X***		
21 CFR 312	***X***		
21 CFR 812		***X***	***X***
21 CFR 820		***X***	

6.0 Procedures Involved*

6.1 The randomized controlled design was chosen because it will minimize bias and identify if a causal relationship exists between the use of laser hair depilation and the rate of pilonidal disease recurrence. See Figure 1. Below (note age range 12-21 has changed):

Figure 1

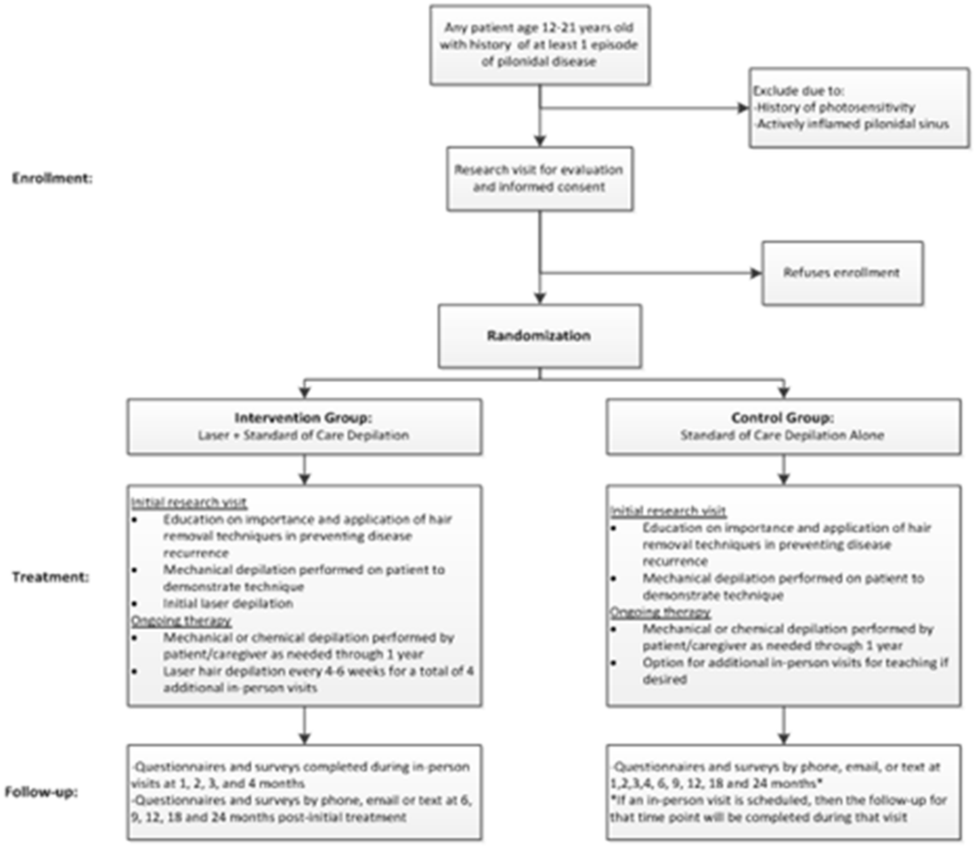

6.2 Laser Depilation plus Chemical/Mechanical Depilation group: Subjects randomized to the laser depilation arm will visit the surgery clinic for 1 treatment every 4-6 weeks to obtain a total of 5 treatments. Fitzpatrick skin type classification will be assessed during the initial visit. The Fitzpatrick skin type classification is based on the level of pigmentation of the skin and its response to ultraviolet light (See Appendix). It will be used to select the best laser to perform hair removal for each patient. The laser treatment group will consist of an 810 nm (for Fitzpatrick skin types I-IV) or Nd:YAG (for Fitzpatrick skin types V-VI) 28 joule application at auto pulse duration for 400 ms. A cooling platform and application of 7% lidocaine/ 7% tetracaine cream, applied 45 minutes prior to treatment, will minimize any discomfort associated with the heat of the laser treatments.

The two lasers that will be used in this study (810 nm or Nd:YAG) are both FDA approved for hair removal in all areas of the human body including the back and perineum. Pilonidal disease is not an FDA approved indication for laser hair removal; however, it is being performed commonly in clinical practice. It was determined, by the Director of the

Drug and Device Development Services and the Institutional Review Board (IRB) at NCH that an investigational device exemption is not necessary because we are using an approved device for investigational purposes with non-significant risk. Laser use in this study poses minimal risk to the patient and these minimal risks are similar to the risks associated with hair removal in other regions of the body for which these devices are already FDA approved. Furthermore, our institutional pilot study confirmed the safety and tolerability of these specific laser machines in an adolescent and young adult population.

Similar to the control group, the patients and families in the intervention group will be taught hair removal techniques and asked to perform either chemical or mechanical depilation as needed to keep the area hair-free between clinic treatments. A protocolized "teach-back" method of patient education will be utilized to confirm knowledge of disease process and treatment. The choice between chemical or mechanical depilation between study visits and after completing the 5th study clinic visit is up to the patient and family based on what they are able to do at home. After the 5th visit, the patient and family will be encouraged to continue to perform regular hair removal in the gluteal cleft as needed until the patient reaches age 30. Razors and depilatory the cream will be provided to each subject with instruction on appropriate use during each clinic visit. After the 5th visit, the patients will need to obtain their own supplies.

Standardized scripts and techniques for both the initial and subsequent education and demonstration of hair removal have been developed and will be utilized for both the control and intervention groups. These have been reviewed by patients, families, and physicians and have been tailored for a Flesch-Kincaid reading level between the 5th and 6th grade with presentation of numbers, percentages, and proportions graphically using figures to minimize the effects of differences in numeracy.

Control; Chemical/Mechanical Depilation group:

Subjects randomized to the control arm will be provided with the "best recommended standard of care" rather than "usual care" in order to ensure that all control subjects receive uniform information and training on mechanical and chemical hair depilation. Therefore, the control group will undergo an initial visit in which they are educated on the importance and application of chronic hair removal. This initial visit will be reflective of what was felt to be a "best practice" clinic visit in which the treating physician performs education and demonstrates the techniques of mechanical and chemical depilation of the gluteal cleft. In addition, the patient and caregiver will be given supplies for hair removal for the next 6 months. This initial visit will provide a standardized level of education and training about hair removal to all patients in the control group. A protocolized "teach-back" method of patient education will be utilized at this visit to confirm knowledge of disease process and treatment. This

method is routinely practiced within our surgical clinic to facilitate effective communication between staff and patients/caregivers. At each monthly phone follow-up through 4 months, patients in the control group will be encouraged to continue to perform regular hair removal in the gluteal cleft until the patient reaches 30 years of age. The importance of hair removal in preventing disease recurrence and the techniques of hair removal will be briefly reviewed in each of these follow-up sessions. In addition, all patients in the control group will have the option to schedule additional in-person visits for further education and training on hair removal. The choice between chemical or mechanical depilation is up to the patient and family based on what they are able to do at home. The potential effects of the control group having fewer in person visits than the intervention group was discussed within our research team. Consensus was that: 1) having the patients in the control group come for an in-person monthly visit for hair removal and clinical exam was neither pragmatic nor reflective of current clinical practice and completely beyond the best standard of care, and 2) having a similar number of monthly follow-up assessments as the intervention group by phone or email would minimize potential negative effects by allowing for similar data collection and reinforcement of the recommendation for continued hair removal.

6.3 *Describe:*

- Attending surgeons and clinical staff in the surgical clinic will be managing the clinical care of the participants. Vital signs, physical exam and pain scores will be assessed per nursing protocol and surgical service standards. An attending surgeon will assess each patient before and after each treatment. A research team call schedule will be maintained with a member of the study team available by pager 24 hours per day. Any suspected adverse event identified by this person will be discussed with the study PI.
- The laser treatment group will consist of an 810 nm (for Fitzpatrick skin types I-IV) or Nd:YAG (for Fitzpatrick skin types V-VI) 28 joule application at auto pulse duration for 400 ms. A cooling platform and application of 7% lidocaine/ 7% tetracaine cream, applied 45 minutes prior to treatment, will minimize any discomfort associated with the heat of the laser treatments.

  The two lasers that will be used in this study (810 nm or Nd:YAG) are both FDA approved for hair removal in all areas of the human body including the back and perineum.

- All baseline and outcome data will be stored electronically in a Research Electronic Data Capture (REDCap) database, which can be accessed only by the appropriate research staff.[32] All

data will be collected in a central REDCap database housed at Nationwide Children's Hospital. To minimize variation and improve the consistency of the interpretation of clinical information, we will utilize standardized data collection forms with established definitions for all variables. See attachment for all surveys/data collected. Although many of our patients return to our institution for subsequent care, some patients may receive care outside of our institution. For patients that receive care outside of our institution, we will capture the events by patient/family report during the follow-up interviews or through medical record review. We will ask for consent from the patient/family and attempt to get the medical records for these encounters to allow us to collect as detailed information about these events as possible.

6.4 Ongoing Assessments

Study staff will conduct data collection at each treatment encounter and at each follow-up time point post- treatment phase. Study staff will contact families with follow-up questions and visit reminders via phone, text, email, and mailed letter, if and when necessary. See attached reminder letter.

In addition, if patients are seen for their pilonidal-related visit at a site outside the NCH network, we will ask participants to sign a release of medical information to obtain visit details regarding the treatment and care they received.

Baseline Assessment

Prior to the first treatment, both the patient and legal guardian will be asked to provide demographic and socioeconomic information (SES) including: age, race, ethnicity, gender, annual household income range, patient occupation (from those who work), guardian occupations and specific insurance coverage status. We will also collect a complete medical history of pilonidal disease, family history of pilonidal disease, current pilonidal disease symptoms, current hygiene regime, and history of physician office visits, Emergency Department/urgent care visits and inpatient hospitalizations for pilonidal disease.

The child and legal guardian will be asked to complete the age appropriate PedsQLTM Quality of Life Inventory and questions about healthcare associated disability, pain and pilonidal disease management. The patient will also complete the Child Attitude Toward Illness Scale (CATIS) and the parent and child will complete the disease-related stigma scales. To assist with the costs and time spent participating in the study, parking

costs will be paid for by the study and a $20 research debit card will be given to the patient or their legal guardian at the end of the visit.

Immediately Following/ 24hrs Post-Treatment (Intervention Group)

Immediately following each laser treatment and 24 hours after, the patient will be asked to rate their pain from 1-10 utilizing the Numeric Rating Scale (NRS-11) (see Appendix for Pain Scale). Contact for the 24 hour follow-up will be completed by phone, text or email.

Follow-up at 1, 2, 3, and 4 months

The patient and legal guardian will complete surveys assessing healthcare associated disability, pain, pilonidal disease management, and Emergency Department/ urgent care/ physician office/hospital visits that occurred between follow-up assessments. In addition, the CATIS will be completed at the 2 month follow-up, the parent and child disease–related stigma scales will be completed at the 3 month follow-up, and the age appropriate Child and Parent PedsQLTM will be administered at the 4 month follow-up. The Healthcare Satisfaction questionnaire will be administered at the 1 and 4 month follow-ups. These surveys and questionnaires will be completed at the in-person visits for patients in the laser group and by either phone or email/web-based survey for patients in the control group (unless they scheduled an in-person visit for any of these follow-ups, in which case they will be completed at the in-person visit). To assist with the costs and time spent participating in the study, a $20 research debit card will be given to the patient or their legal guardian at the end of the visit or mailed to the legal guardian's address (or patient's address if 18 years of age or older) upon successful completion of the surveys.

Subjects who will turn 18 before their 1, 2, 3, and 4 month follow-ups will be asked to sign the informed consent used for the study and to update their contact information. After speaking with the subject and explaining the study, an informed consent and contact information sheet will be mailed to the subjects to sign so that we may continue to collect data up to 24 months after their initial study treatment.

Post-Treatment Follow-Ups at 6, 9, 12, 18, and 24 months

Each subject will receive an email or telephone call at 6, 9, 12, 18, and 24 months after their initial visit. Follow-up will be conducted by email/web-based survey or by phone with a member of the research team from NCH. The patient and legal guardian will complete surveys assessing healthcare associated disability, pain, pilonidal disease management, and Emergency Department/ urgent care/ physician office/hospital visits that occurred between follow-up assessments. At 6 and 12 months, the age appropriate

Child and Parent PedsQLTM and the CATIS will be administered. At 9 months, healthcare satisfaction and disease related stigma scales will be administered. A $20 research debit card will be mailed to the legal guardian's address (or patient's address if ≥18 years of age) upon successful completion of the surveys at 6 and 9 months. A $50 research debit card will be mailed the legal guardian's address (or patient's address if ≥ 18 years of age) upon successful completion of the surveys at 12 months.

At the 12 month time point, patients randomized to the control group will be offered laser treatment, at no cost, contingent on the interim analyses of adverse events for this study. If these patients choose to receive the elective laser treatments, they will be brought back into the clinic and re-consented by the study staff prior to their first treatment. They will have the option to commit to one or two additional years in the study and will be followed in the same manner as the patients initially randomized to the laser intervention group. Data will be collected from the patients per the same follow-up surveys at 1, 2, 3, and 4 months and post-treatment follow-up surveys at 6, 9, 12, 18, and 24 months after their initial visit. A $20 research debit card will be mailed to the legal guardian's address (or patient's address if ≥ 18 years of age) upon successful completion of the surveys at 6and 9 months. A $50 research debit card will be mailed the legal guardian's address (or patient's address if ≥ 18 years of age) upon successful completion of the surveys at 12 months. Since laser therapy has previously been shown to be safe and tolerable, patients and caregivers in the control group will be informed of the safety monitoring data and the recurrence rates from the interim analysis when available (Analysis section above) and allowed to decide if they would like to receive laser therapy.

Subjects who will turn 18 before their 12, 18, or 24 month follow-up will be asked to sign the informed consent used for the study and to update their contact information. After speaking with the subject and explaining the study, an informed consent and contact information sheet will be mailed to the subjects to sign so that we may continue to collect data up to 24 months after their initial study treatment.

6 months post 1st treatment (±30 days)

A member of the research team from NCH will contact the family over the phone or email and ask questions about any problems the child has had with their pilonidal disease since their 30-day follow-up. Specifically, we will ask about subsequent ED/Urgent Care visits or physician visits, readmissions and operations, additional imaging, and missed days from normal activities by the patient and legal guardian that were related to their pilonidal disease. We will also ask about the out-of-pocket

expenditures directly spent on post-treatment medical care and returning visits (e.g. insurance copayments; costs of drugs) and other expenditures related to the visits (e.g. transportation costs) within one year. Please see the form in the appendix for data points to be collected during this phone call.

For subjects who will turn 18 before their 6-month follow-up, they will be asked to sign the informed consent used for the study and to update their contact information. After speaking with the subject and explaining the study, an informed consent and contact information sheet will be mailed to the subjects to sign so that we may continue to collect data up to 24 months after their initial study treatment.

A $20 research debit card will be mailed to the legal guardian's address upon successful completion of the survey.

9 months (±30 days)

A member of the research team from NCH will call or email the family and ask questions about any problems the child has had with their pilonidal disease since their six-month follow-up. Specifically, we will ask about subsequent ED/Urgent Care visits or physician visits, readmissions and operations, additional imaging, and missed days from normal activities by the patient and legal guardian that were related to their pilonidal disease. We will also ask about the out-of-pocket expenditures directly spent on post-treatment medical care and returning visits (e.g. insurance copayments; costs of drugs) and other expenditures related to the visits (e.g. transportation costs) within one year. Please see the form in the appendix for data points to be collected during this phone call.

For subjects who will turn 18 before their 9-month follow-up, they will be asked to sign the informed consent used for the study and to update their contact information. After speaking with the subject and explaining the study, an informed consent and contact information sheet will be mailed to the subjects to sign so that we may continue to collect data up to 24 months after their initial study treatment.

A $20 research debit card will be mailed to the legal guardian's address upon successful completion of the survey.

12 months post 1st treatment (±30 days)

A member of the research team from NCH will call or email the family and ask questions about any problems the child has had with their pilonidal disease since their 9-month follow-up. Specifically, we will ask about subsequent ED/Urgent Care visits or physician visits, readmissions

and operations, additional imaging, and missed days from normal activities by the patient and legal guardian that were related to their pilonidal disease. We will also ask about the out-of-pocket expenditures directly spent on post-treatment medical care and returning visits (e.g. insurance copayments; costs of drugs) and other expenditures related to the visits (e.g. transportation costs) within one year. Please see the form in the appendix for data points to be collected during this phone call. For patients that are unresponsive to follow up, we will contact their primary care physician (PCP) to assess event related to their pilonidal disease.

For subjects who will turn 18 before their 12-month follow-up, they will be asked to sign the informed consent used for the study and to update their contact information. After speaking with the subject and explaining the study, an informed consent and contact information sheet will be mailed to the subjects to sign so that we may continue to collect data up to 24 months after their initial study treatment.

A $50 research debit card card will be mailed to the legal guardian's address for the completion of this time point.

*If at the 12 month time point, laser depilation is shown to be effective, patients randomized to the control group will be offered laser treatment at no cost. Patients choosing to receive laser treatments at no cost will be also asked to re-consent at this time and will be asked to complete surveys at the 1, 2 , 3, 4, 6, 9, and 12 months follow-up time points. However, only patients will only receive $20 for the completion of the 6 and 9month survey completions, and a $50 for completion of the surveys at 12 months. As we are asking this group of standard care to laser care patients to continue to fill out surveys and attend laser visits (in the same fashion as the group originally randomized to laser) we will ask these patients to provide an additional consent at the time that they begin to receive laser treatments.

* If at the 12 month time point, the subject treated with the laser feel that they have significant hair re-growth, they will be asked if they would like to be re-evaluated for further laser hair removal at no cost.

18 and 24 months post 1st treatment (±30 days)

A member of the research team from NCH will call or email the family and ask questions about any problems the child has had with their pilonidal disease since their 12 or 24-month follow-up. Specifically, we will ask about subsequent ED/Urgent Care visits or physician visits, readmissions and operations, additional imaging, and missed days from normal activities by the patient and legal guardian that were related to their pilonidal disease. We will also ask about the out-of-pocket

expenditures directly spent on post-treatment medical care and returning visits (e.g. insurance copayments; costs of drugs) and other expenditures related to the visits (e.g. transportation costs) within one year. Please see the form in the appendix for data points to be collected during this phone call.

For subjects who will turn 18 before their 12 or 24-month follow-up, they will be asked to sign the informed consent used for the study and to update their contact information. After speaking with the subject and explaining the study, an informed consent and contact information sheet will be mailed to the subjects to sign so that we may continue to collect data up to 24 months after their initial study treatment.

Participants will not be reimbursed for the 18 and 24 month follow-up time points.

COVID-19 additional surveys

Patients whose treatments are delayed, may still complete the 1, 2, 3, and 4 month follow up surveys without being seen for their laser treatments. When the non-essential visits open back up, patients will complete the rest of their treatments and complete the corresponding visit surveys. Since these are additional surveys that were not a part of the original consent, the patient will be asked to voluntarily sign an addendum demonstrating their willingness to complete the surveys. These additional surveys will replicate the corresponding visit surveys previously mentioned. To assist with the costs and time spent participating in the study, a $20 research debit card will be given to the patient or their legal guardian at the end of the visit or mailed to the legal guardian's address (or patient's address if 18 years of age or older) upon successful completion of the surveys.

6.5 If there are plans for long-term follow-up (once all research related procedures are complete), what data will be collected during this period. N/A

6.6 For Humanitarian Use Device (HUD) uses provide a description of the device, a summary of how you propose to use the device, including a description of any screening procedures, the HUD procedure, and any patient follow-up visits, tests or procedures. N/A

7.0 Data and Specimen Banking*

7.1 All demographic, clinical, and outcome data will be recorded by trained study staff in the REDCap database maintained at Nationwide Children's Hospital. REDCap is a network software suite that provides data storage, encryption, and password protection for health information, to which only study staff will have access. Privacy and security will be maintained by minimizing the amount of identifiable data collected. Local

data will be stored on computers located within The Center for Surgical Outcomes Research (611 East Livingston Avenue, Columbus, OH, 43206) facilities that are maintained on secure networks. All data and records generated during this study will remain confidential. All documents will be used solely for this study. De-identified data will be stored indefinitely as part of a repository.

7.2 MRN, Name, Phone Number, MyChart User or Not, Home Address, Email ID, Age, Race, Ethnicity, Gender, Insurance Coverage Status, Socioeconomic Data, Medications, Labs, Medical History Related to Pilonidal Disease, Family History of Pilonidal Disease, Current Pilonidal Disease Symptoms, Physician Office Visit, Emergency/Urgent Care Visit, Inpatient Visits for Pilonidal Disease

7.3 Privacy and security will be maintained by minimizing the amount of identifiable data as much as possible. Only study identifications (IDs) will be used to identify patients on all data forms and all datasets used for analysis. The file linking study IDs to patient names and medical record numbers (MRNs) will be password protected and will not be made available to non-study staff or used during data analysis. All study information will be compiled in REDCAP, to which only study staff will have access. There are no plans at this time to use the data collected in this study for a future research. In the event that a comparative study is planned in the future we will apply for approval to retrospectively review all procedures used for the treatment of pilonidal disease, which would include the patient population from this study amongst others.

8.0 Sharing of Results with Subjects*

8.1 The final study results will not be shared with participants individually. However, at some time, a final study summary will be available on the ClinicalTrials.Gov (http://clinicaltrials.gov) website.

9.0 Study Timelines*

9.1 *Describe:*

- The duration of an individual subject's participation in the study is two years.
- Anticipated enrollment will be completed 09/01/2021.
- Estimated completion of final submission to grant is 8/15/2023.

10.0 Inclusion and Exclusion Criteria*

10.1 All potentially eligible patients treated for pilonidal disease at NCH, OSU, or one of the community pediatric practices will be informed of the study. If they do want more details, they will ask for permission to provide their contact information to the study team. Subsequently, a trained member of the research team will contact the patient and family to assess their eligibility against both inclusion and exclusion criteria. If all

eligibility criteria are met, the patient and legal guardian (if < 18 years of age) will be invited to enroll. At the initial clinic visit, a trained member of the research team will confirm the patient is eligible.

10.2 Inclusion Criteria

- All Fitzpatrick skin types
- Age: 11-21 years
- Previous diagnosis or concern for active pilonidal disease

Exclusion Criteria

- History of photosensitivity
- Actively inflamed pilonidal sinus. These patients will be informed of the trial and invited to contact the study team upon resolution of their inflamed sinus if they are interested in being in the trial at that time.

10.3 Our study will include children and teenagers. It will exclude adults unable to consent, pregnant women, and prisoners.

11.0 Vulnerable Populations*

11.1 The study will be conducted within a pediatric surgery clinic and will therefore involve minors. As a pediatric research hospital we will continue to utilize the stringent privacy safeguards that are in place for all patients treated at this institution. This study falls under Section 2 or HRP 416 as it is greater than minimal risk to the participants.

12.0 Local Number of Subjects

12.1 The sample size needed to assess the primary outcome of the proportion of patients with recurrent pilonidal disease at 1-year is based on: 1) previous published studies and institutional data on the recurrence rates of pilonidal disease and 2) the efficacy of laser hair depilation treatment to reduce pilonidal disease recurrence in previous studies and our institutional pilot study. In this RCT, the recurrence rate within 1 year is expected to be 12% in the control group and a maximum 2% in the laser group. Based on these estimated recurrence rates, under a group sequential design with one interim and one final analysis, an overall type I error rate (two- sided) of 5 % and power of 80%, the sample size required for this trial is 122 patients in each treatment group. Assuming a 10% drop out rate over the course of the 1 year follow-up, we will plan to enroll 136 patients in each group (total; n=272 patients).

The gap in the laser treatments caused by COVID-19 created a gap in when we could begin laser treatments in newly enrolled patients by 1-3 months. Since a laser patient's participation in the study begins at their first laser treatment, no data has been collected on these patients. Patients in the group have reported no longer being interested/able to receive

treatments due to concerns about COVID and visits to the hospital to receive laser treatments. This group of about 15 patients was not anticipated for our sample size calculation but are needed for the primary outcomes data. In order to maintain the required power in our analyses, our statistician recommends enrolling and randomizing an additional 30 patients, thereby increasing our sample size to 302 patients (151 in each group).

12.2 If applicable, distinguish between the number of subjects who are expected to be enrolled and screened, and the number of subjects needed to complete the research procedures (i.e., numbers of subjects excluding screen failures.) -N/A

13.0 Recruitment Methods

13.1 All potentially eligible patients treated for pilonidal disease at NCH, OSU, or one of the community pediatric practices will be informed of the study. All potential patient recruitment sites will have flyers and pamphlets advertising the study. Study team members will contact sites monthly to answer questions and provide additional information and study related materials as needed. Clinic schedules will be reviewed by members of the research team and providers seeing patients with pilonidal disease will be asked if they can be approached about the study. Nurses in each of the clinics will be informed of the study and inform patients about the study and provide study contact information to the patient and their family. In addition, they will ask the patient and family if they would like additional information on the study. If they do want more details, they will ask for permission to provide their contact information to the study team. Subsequently, a trained member of the research team will contact the patient and family to assess their eligibility against both inclusion and exclusion criteria. If all eligibility criteria are met, the patient and legal guardian (if < 18 years of age) will be invited to enroll. The research team member will review the information about the study and answer any questions. If they remain interested in enrolling, they will be scheduled for a clinic visit. At the initial clinic visit, a trained member of the research team will review the study protocol and procedures and the risk and benefits of each treatment with the patient and caregiver again, answer any additional questions, and written informed consent and assent (for patients < 18 years of age) will then be obtained.

If a patient is enrolled into the study at a telehealth visit, a trained member of the research team will contact the family to obtain phone or electronic-consent. At the end of the telehealth video visit, if the patient verbally states they are interested in the study, a trained member of the study will join the telehealth call through EPIC by invite of the principal investigator. The trained member will review the study protocol and procedures and the risk and benefits of each treatment with the patient and caregiver, answer any questions, and phone/ electronic-consent and assent (for patients < 18

years of age) will then be obtained. If the patient uses electronic-consent, it will be given via REDCap. A signed copy of the consent will be sent to the patients. Due to COVID-19 restrictions, there may be a delay in mailing the signed consent forms. However, when social restrictions are lifted and staff are able to access the necessary resources, the consent will be mailed.

13.2 The study will be conducted at Nationwide Children's Hospital (NCH). Study participants will be recruited in the NCH surgery clinic (in-person or through telehealth), ED, inpatient units and by phone (for patients interested in the study from The Ohio State University Wexner Medical Center, community pediatric and adult practices, emergency rooms and urgent cares, community recreation boards, local colleges, local high schools, Columbus City Schools, and all those employed or seeking care through the NCH network – see Section 13.3).

13.3 All potentially eligible patients treated for pilonidal disease at NCH, The Ohio State University Wexner Medical Center (OSU), or one of the community pediatric and adult practices will be informed of the study. Potential participants may also be identified through numerous outreach efforts conducted through brochures and flyers made available at emergency rooms and urgent cares, community recreation boards, local colleges, local high schools, Columbus City Schools, and all those employed or seeking care through the NCH network.

If interested, their names and contact information will be sent to the research team at NCH by a provider or referral source or those receiving study information via one of the informational outlets will contact a member of the study team directly for additional information.

In addition, a bi-monthly automated data report will be generated for the study staff to help identify all patients seen in the NCH network and at The Ohio State University Wexner Medical Center with a diagnosis of pilonidal disease. Potential candidates identified through these data reports will be sent a letter and brochure describing the study and contact information via snail mail.

As part of OSU Wexner Medical Center Recruitment, this study has been invited to participate in a recruitment effort through OSU's MyChart Portal.

Ohio State's digital health portal, MyChart, is a system that enables patients to connect with their healthcare providers and manage their health information through a variety of interactive functions. The MyChart Recruitment function is a new recruitment approach for researchers that operates through the MyChart message inbox. This recruitment approach will limit unnecessary study communication by targeting messages to patients that are eligible based on study inclusion criteria. This message will allow the patient to express interest in study to allow for a research

team member to make contact with them, contrarily, the patient also has the opportunity to decline subsequent information, at which time no further contact will be made with that patient concerning the study. Our study has been selected as one of four pilots for the launch of this function in the beginning of February.

In addition to the automated, introductory message, the potential participant will receive an attached description of the study protocol (study message). This messaging can be found in the appendix attached.

Here is how we will identify and notify potential study participants through OSU MyChart Recruitment:

i. All the PHI that is required for the study – eg: MRN, MyChart User or Not, Phone number, Home Address, Email Id, Contact Info, Demographics, Medication, Labs, Any Dates of interest etc.

MRN, Name, Phone Number, MyChart User or Not, Home Address, Email ID, Age, Race, Ethnicity, Gender, Insurance Coverage Status, Socioeconomic Data, Medications, Labs, Medical History Related to Pilonidal Disease, Family History of Pilonidal Disease, Current Pilonidal Disease Symptoms, Physician Office Visit, Emergency/Urgent Care Visit, Inpatient Visits for Pilonidal Disease (Same PHI for non-MyChart Users defined in aforementioned sections of protocol).

ii. The conditions/eligibility criteria to identify cohort.

Eligibility criteria include patients with:

Fitzpatrick skin types, 11-21 years of age, and a previous diagnosis and treatment for active pilonidal disease. To identify the cohort through OSU MyChart, patients will be identified in the query through the Information Warehouse that fit the study inclusion criteria by an Honest Broker. The query will include a combination of CPT codes and ICD-10 codes listed as follows:

ICD-10 Codes: LO5.01, LO5.02, LO5.91, LO5.92
ICD-10-PCS Codes: 0H96XZZ, 0JB90ZZ, 0H98XZZ, 0HX8XZZ
CPT Codes: 01999, 10090, 10081, 11770, 11771, 11772, 88304

iii. A list of MRNs (obtained through IW) will go directly to Research IHIS team (Nicole Rutledge) and the Research IHIS team will use the identified MRNs to send messages via MyChart . This list of patients and their contact information will also be shared with the NCH study team so an additional letter can be sent to the patient's home to notify them that they may be eligible for our study.

iv. (Only) For the Patients who said "Contact Me" (accepted) the medical record will be viewable by study team.

v. Script (written or spoken) to be the used for communications through MyChart with interested patient. Each message sent through MyChart is automatically introduced with the following message by the system:

A computer-generated search found that you may fit the study described below. Unless you decide to click interested in this research opportunity, no one on the research team will know that you have been contacted. Your privacy and your health are most important to us. The Ohio State Wexner Medical Center is driven by our mission “to improve people’s lives through innovation in research, education, and patient care.

If the patient indicates that he/she is interested in the study, a research member will reply to the patient with: Thank you for your interest in the Pilonidal Laser Hair Removal Study. A trained member from our research team will be in contact with you shortly to discuss the details of the study and answer any questions or concerns you may have. If there is a preferred contact number, you may reply to this message with that information. If not, we will use the preferred contact information found in your chart. We look forward to talking to you soon. Thank you.

Pilonidal Research Team

Subsequently, a member of the research team will contact all identified patients received via data report, provider referral, and outreach outlets to assess their eligibility against both inclusion and exclusion criteria. If all eligibility criteria are met, the patient and legal guardian (if < 18 years of age) will be invited to enroll. The research team member will review the information about the study and answer any questions. If they remain interested in enrolling, they will be scheduled for a clinic visit. At the initial clinic visit, a member of the research team will (1) review the study protocol and procedures; (2) explain the risk and benefits of each treatment; (3) answer questions; and (4) obtain written informed consent and assent (for patients 11-17 years of age). All of the enrollment materials have been reviewed by patients, families, pediatricians, and surgeons to ensure that they are balanced representations of the risks and benefits of each treatment option. These materials have been tailored for a Flesch-Kincaid reading level between the 5th and 6th grade to ensure that both children and low-literacy adults are able to understand them. In addition, we have attempted to minimize the effects of differences in numeracy by presenting numbers, percentages, and proportions graphically using figures to facilitate understanding. A member of the research team will be available by pager 24 hours per day to answer and address study-related questions from patients, families, and referring medical providers.

13.4 All potential patient recruitment sites will have flyers and pamphlets advertising the study. See attached marketing documents.

13.5 *Describe the amount and timing of any payments to subjects.*

14.0 Withdrawal of Subjects*

14.1 If the Principal Investigator believes that this study is not good for the participant, the study instructions are not followed, participation in the study may be stopped. If unexpected medical problems come up, the Principal Investigator may decide to also withdraw the participant from the study.

14.2 Participants and their legal guardian have the right to switch to the standard treatment and/or withdraw from the study at any time. Withdrawal from the study will not affect receipt of clinical care. If the participant is terminated, the study will contact the participant and inform them,

14.3 If the participant withdraws participation study members will ask permission to retain authorization of the use of their PHI. If they want to revoke that permission then they must request this in writing to the PI at Nationwide Children's Hospital, 700 Children's Drive, Columbus, OH 43205. If the participant withdraws their authorization, no new PHI will be collected and the PHI already collected will not be used unless it has already been used or is needed to complete the study analysis and reports.

15.0 Risks to Subjects*

15.1 Laser Depilation Group Potential risks may include:

- No relief of symptoms
- Recurrence of pilonidal abscess or wound complication
- Photosensitivity or discomfort from laser treatments
- Lastly, loss of confidentiality could be a risk.

Chemical/Mechanical Depilation group:

- Loss of confidentiality could be a risk.

We believe that there is very little risk as a result of being in this study.

Laser treatment may cause some pain or discomfort during and after the laser treatment. We will be applying a topical anesthetic to the skin prior to the laser treatment and will monitor the skin closely for any significant redness or tenderness after the treatment. Trained laser operators will be in the room to make sure the laser is working safely so as to minimize any risks.

The topical anesthetic cream may cause a burning feeling on the skin, a change in color of the skin in that area, or can temporarily affect the way your blood carries oxygen, called methemoglobinemia. We will watch for any of these problems and then check on the area to see if the infection has returned or if it has gotten better as the treatments happen.

It is possible that the participant could feel upset when answering questions about your diagnosis or medical treatment, but it may be more likely that you find the questions or feedback process a little boring.

Although we will take every precaution, there is a small chance of loss of confidentiality of PHI.

15.2 If applicable, indicate which procedures may have risks to the subjects that are currently unforeseeable.-N/A

15.3 If applicable, indicate which procedures may have risks to an embryo or fetus should the subject be or become pregnant. – N/A

15.4 If applicable, describe risks to others who are not subjects. -N/A

16.0 Potential Benefits to Subjects*

16.1 Laser Depilation Group Potential benefits may include:

- Faster relief of symptoms (ex: resolution of pain, erythema)
- Avoiding surgery altogether
- No risk of surgical complications
- Possible decrease in folliculitis or recurrence

Chemical/Mechanical Depilation Group

- Possible decrease in folliculitis or recurrence due to better hair removal from multiple clinic visits
- Receipt of laser therapy upon completion of 1 year follow-up at no charge if it is shown to be effective.

Laser depilation treatments will be provided at no cost to the patient. All clinic visits and medications related to the study will be provided at no cost to the patient. Patients randomized to the control group will be offered laser therapy upon completion of 1 year follow-up at no charge if it is shown to be effective.

To assist with the costs and time spent participating in the study, a $20 research debit card will be given to the patient or their legal guardian at the end of the visit or mailed to the legal guardian's address (or patient's address if <18 years of age) upon successful completion of the surveys. After initial follow up, a $20 research debit card will be mailed to the legal guardian's address (or patient's address if <18 years of age) upon successful completion of the surveys at 6 and 9 months. A $50 research debit card will be mailed the legal guardian's address (or patient's address if <18 years of age) upon successful completion of the surveys at 12 months. Participants will not receive payment for the completion of the 18 and 24 month time points.

16.2 Indicate if there is no direct benefit. Do not include benefits to society or others. – N/A

17.0 Data Management* and Confidentiality

17.1 The means/medians and standard deviations/interquartile ranges of the prognostic variables will be evaluated in the total study sample and will be compared between groups using t-tests or Mann Whitney U tests for continuous variables and chi-squared tests for categorical variables. For the primary outcome of recurrence within one year, we will calculate this proportion and its 95% confidence interval, based on the exact binomial distribution, in both treatment groups and compare this proportion between groups using a Chi-squared test.

Other outcomes will be compared using chi square tests, if categorical, and t-tests or Mann Whitney U tests as appropriate if continuous. In analyses that include outcome data collected after patients in the control group were offered laser treatment, both intention-to-treat and per-protocol analyses (with control group patients analyzed according to the treatment they received and according to the time from the start of their laser treatment) will be performed. SAS Enterprise Guide (Cary, NC) will be used for the statistical analyses.

The sample size needed to assess the primary outcome of the proportion of patients with recurrent pilonidal disease at 1-year is based on: 1) previous published studies and institutional data on the recurrence rates of pilonidal disease and 2) the efficacy of laser hair depilation treatment to reduce pilonidal disease recurrence in previous studies and our institutional pilot study. In this RCT, the recurrence rate within 1 year is expected to be 12% in the control group and a maximum 2% in the laser group. Based on these estimated recurrence rates, under a group sequential design with one interim and one final analysis, an overall type I error rate (two- sided) of 5 % and power of 80%, the sample size required for this trial is 122 patients in each treatment group. Assuming a 10% drop out rate over the course of the 1 year follow-up, we will plan to enroll 136 patients in each group (total; n=272 patients). The total of patients increased to 302 due to reasons related to COVID described above.

Heterogeneity of treatment effects (HTE) will be formally explored for four characteristics: episodes of previous of disease, previous surgical excision performed, gender, and BMI (Figure 2: Conceptual Model). However, as the evidence regarding the effects of these factors on recurrence rates is not consistent in the literature, these HTE analyses are considered exploratory and the study will not be powered specifically for analyses of treatment effects in these subgroups

Figure 2: Conceptual Model

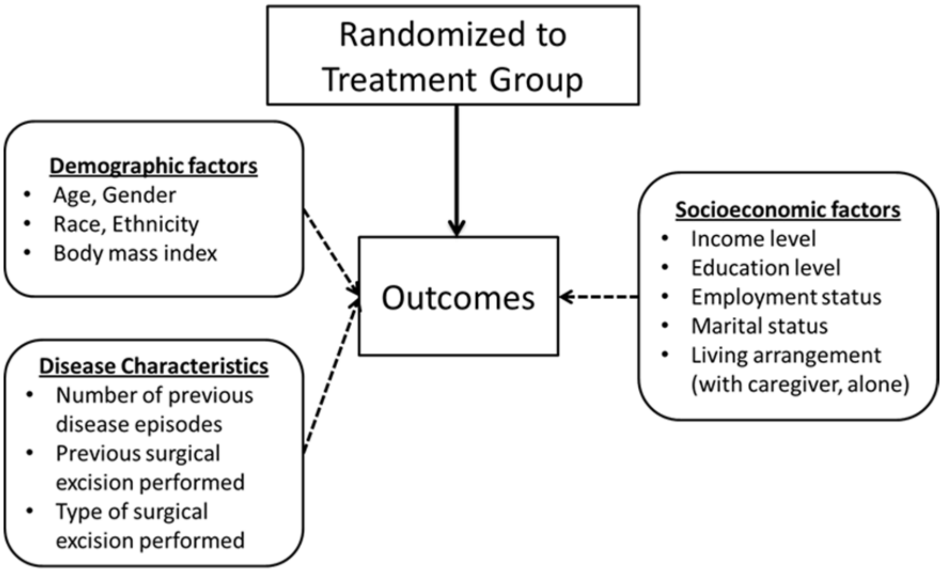

17.2 Patients that consent to enroll will be randomized using a randomized block scheme, with blocks of varying size. The sequence will be built using the plan procedure in SAS 9.4 (SAS Institute Inc., Cary, NC). It will be generated and maintained by the project statistician and will be unavailable to other research study members. Blocks will be of size four or six, with the lengths chosen randomly with equal probability. This block design assures a balanced allocation to the two treatment groups, both at the end of trial enrollment and at periodic intervals throughout enrollment. The varying block size also reduces the chance that research personnel will be able to guess the next intervention group assignment and, thus, minimizes any unconscious bias in patient allocation. Stratification will not be incorporated into the randomization scheme because, based on the current literature, there are no patient characteristics, including the number of previous episodes of disease, the types of previous treatments, or disease severity, that are consistently strongly associated with the probability of recurrence. Furthermore, all subgroups defined by these characteristics are expected to be large enough that the risk of imbalance in these characteristics is unlikely, though this will be monitored.

A web-based randomization and registration process will be designed to verify inclusion/exclusion criteria and then randomize eligible patients (using the earlier generated randomization list). These data will reside behind the Nationwide Children's Hospital (NCH) firewall for security. All data including the registration and randomization will be backed up incrementally daily with a full backup weekly. Tracking and error reports will be generated to alert study staff of any data quality issues as they arise. Research staff will follow up on any instances of incomplete or problem data. All baseline and outcome data will be stored electronically

in a Research Electronic Data Capture (REDCap) database, which can be accessed only by the appropriate research staff. Given the nature of the intervention proposed, participants and research team members will know the intervention to which they have been assigned.

17.3 See sections 17.1 and 17.2

17.4 Describe how data or specimens will be handled study-wide:

- MRN, Name, Phone Number, MyChart User or Not, Home Address, Email ID, Age, Race, Ethnicity, Gender, Insurance Coverage Status, Socioeconomic Data, Medications, Labs, Medical History Related to Pilonidal Disease, Family History of Pilonidal Disease, Current Pilonidal Disease Symptoms, Physician Office Visit, Emergency/Urgent Care Visit, Inpatient Visits for Pilonidal Disease
- Privacy and security will be maintained by minimizing the amount of identifiable data as much as possible. Only study identifications (IDs) will be used to identify patients on all data forms and all datasets used for analysis. The file linking study IDs to patient names and medical record numbers (MRNs) will be password protected and will not be made available to non-study staff or used during data analysis.
- All study information will be compiled in REDCAP, to which only study staff will have access.
- De-identified data will be stored indefinitely as part of a repository. Identified data will be kept for 6 years.
- All data and records generated during this study will remain confidential. All documents will be used solely for the use of this study by approved personnel. Consent forms and written surveys will be maintained at NCH in locked cabinets and all other data will be maintained in a central REDcap database that would be housed at NCH for 6 years. De-identified data exported from REDCap for statistical analysis will be maintained in password-protected files and on password-protected computers at NCH.

- Who is responsible for receipt or transmission of the data or specimens? N/A
- How data or specimens will be transported? N/A

18.0 Provisions to Monitor the Data to Ensure the Safety of Subjects*

This section is required when research involves more than Minimal Risk to subjects.

18.1 Describe: N/A

19.0 Provisions to Protect the Privacy Interests of Subjects

19.1 Study members will only contact participants a maximum of three times for recruitment and follow up surveys. Please see section 13 for additional information.

19.2 The patient is informed from the beginning that participation is voluntary and they may withdraw at any time. They also are not required to answer any survey question that they do not want to. This is laid out clearly in the consent form.

19.3 Sources for data collection will be the patients' electronic health record (EPIC) and REDCap suvreys that will be stored in the REDCap database.

20.0 Compensation for Research-Related Injury

20.1 If the research involves more than Minimal Risk to subjects, describe the available compensation in the event of research related injury. – N/A

20.2 Language included in the protocol: "Based on previous experience, it is unlikely that you will experience any serious problems as a result of this study.

If you are hurt by the study procedures that are part of this study, you should seek medical treatment for the injuries and call the study team as soon as possible at the number on page 1 of this form. If it is an emergency, call 911 or go to the nearest emergency department.

In most cases, this care will be billed to your health insurance company or whoever usually pays for your health care at the usual charges, but some insurance companies will not pay for care related to a study. If the care is provided at Nationwide Children's Hospital or The Ohio State University, we make no commitment to pay for the medical care provided to you. No funds have been set aside to compensate you in the event of injury. If no one else pays for your care, you may have to pay for the cost of this care. This does not mean that you give up any of your legal rights to seek compensation for your injuries."

21.0 Economic Burden to Subjects

21.1 All costs related to the research parts of this study will be covered by the research team. However, the parts of the study that would be done for routine clinical care will be billed to the participant and to their insurance company or third party payer. The participant may have to pay any costs that the insurance company or third party payer does not pay. There will be additional costs related to travel and meals during this study.

22.0 Consent Process

22.1 *Indicate whether you will you be obtaining consent, and if so describe:*

- Informed consent will be obtained on the main campus of NCH, close to home centers, or via telehealth.
- Waiting period is contingent on the participants need.
- Any process to ensure ongoing consent. N/A
- *Our study will be following "SOP: Informed Consent Process for Research (HRP-090*

Non-English Speaking Subjects – if known, skip if not known

- *Indicate what language(s) other than English are understood by prospective subjects or representatives.*
- *If subjects who do not speak English an interpreter will be present for the clinic visit. A Spanish consent is available for those who speak Spanish.*

Waiver or Alteration of Consent Process (consent will not be obtained, required information will not be disclosed, or the research involves deception)

- Review the "CHECKLIST: Waiver or Alteration of Consent Process (HRP-410)" to ensure you have provided sufficient information for the IRB to make these determinations.
- If the research involves a waiver the consent process for planned emergency research, please review the "CHECKLIST: Waiver of Consent for Emergency Research (HRP-419)" to ensure you have provided sufficient information for the IRB to make these determinations. N/A

Waiver of Written Documentation of Consent (verbal)

- Review the CHECKLIST: Waiver of Written Documentation of Consent Process (HRP-411) to ensure you have provided sufficient information for the IRB to make these determinations. N/A

Subjects who are not yet adults (infants, children, teenagers)

- If the child and legal guardian (for subjects ≥11 and < 18 years) are interested in participating in this study, a physician-member of the research team will guide the child and legal guardian through the informed consent/assent process. Written informed consent will be obtained from one legal guardian of subjects ≥11 and

- < 18 years of age and from the patient him or herself if he or she is ≥ 18 years of age. Written informed assent will be obtained from subjects ≥11 and < 18 years of age.

Cognitively Impaired Adults

- *Describe the process to determine whether an individual is capable of consent. The IRB allows the person obtaining assent to document assent on the consent document and does not routinely require assent documents and does not routinely require children to sign assent documents. N/A*

Adults Unable to Consent

- List the individuals from whom permission will be obtained in order of priority. (E.g., durable power of attorney for health care, court appointed guardian for health care decisions, spouse, and adult child.)
  - For research conducted in the state, review "SOP: Legally Authorized Representatives, Children, and Guardians (HRP-013)" to be aware of which individuals in the state meet the definition of "legally authorized representative."
  - For research conducted outside of the state, provide information that describes which individuals are authorized under applicable law to consent on behalf of a prospective subject to their participation in the procedure(s) involved in this research. One method of obtaining this information is to have a legal counsel or authority review your protocol along the definition of "legally authorized representative" in "SOP: Legally Authorized Representatives, Children, and Guardians (HRP-013)."
- Describe the process for assent of the subjects. Indicate whether:
  - Assent will be required of all, some, or none of the subjects. If some, indicated, which subjects will be required to assent and which will not.
  - If assent will not be obtained from some or all subjects, an explanation of why not.
  - Describe whether assent of the subjects will be documented and the process to document assent. The IRB allows the person obtaining assent to document assent on the consent document and does not routinely require assent documents and does not routinely require subjects to sign assent documents. N/A

Adults Unable to Consent

- For HUD uses provide a description of how the patient will be informed of the potential risks and benefits of the HUD and any procedures associated with its use. N/A

23.0 Process to Document Consent in Writing

23.1 Our study is following the "SOP: Written Documentation of Consent (HRP-091)."

24.0 Setting

24.1 *Describe the sites or locations where your research team will conduct the research.*

- Research personnel will be housed at the Center for Surgical Outcomes Research at Nationwide Children's Hospital (611 E. Livingston Avenue, Columbus, OH 43206). Recruitment will happen at NCH Main Campus, NCH Close to Home, or via telehealth. See details in section 13.
- Procedures will occur on the 6th floor of the OCC at NCH Main Campus.
- Our study has a stakeholder group made up of physicians, nurses, and patients. Additionally, we work with a DSMB.
- For research conducted outside of the organization and its affiliates describe: N/A
    - Site-specific regulations or customs affecting the research for research outside the organization.
    - Local scientific and ethical review structure outside the organization.

25.0 Resources Available

25.1 *Describe the resources available to conduct the research: For example, as appropriate:*

Dr. Minneci has an established an outpatient clinic at Nationwide Children's Hospital dedicated to treating pediatric patients with pilonidal disease. In a typical month, approximately 60 patients will be seen and evaluated in this clinic. The resources needed to obtain eligible patients are readily available and the clinical pathway has been established and clinical decisions will not be made based off this study.

26.0 Multi-Site Research*

26.1 *Study-Wide Number of Subjects** N/A
If this is a multicenter study, indicate the total number of subjects to be accrued across all sites.

27.0 Protected Health Information Recording

1.0 Indicate which subject identifiers will be recorded for this research.

☒ Name
☒ Complete Address
☒ Telephone or Fax Number
☐ Social Security Number (do not check if only used for ClinCard)

☒ Dates (treatment dates, birth date, date of death)
☒ Email address, IP address or url
☒ Medical Record Number or other account number
☐ Health Plan Beneficiary Identification Number
☐ Full face photographic images and/or any comparable images (x-rays)
☐ Account Numbers
☐ Certificate/License Numbers
☐ Vehicle Identifiers and Serial Numbers (e.g. VINs, License Plate Numbers)
☐ Device Identifiers and Serial Numbers
☐ Biometric identifiers, including finger and voice prints
☐ Other number, characteristic or code that could be used to identify an individual
☐ None (Complete De-identification Certification Form)

2.0 Check the appropriate category and attach the required form* on the Local Site Documents, #3. Other Documents, page of the application. (Choose one.)

☒ Patient Authorization will be obtained. (Include the appropriate HIPAA language (see Section 14 of consent template) in the consent form OR attach the HRP-900, HIPAA AUTHORIZATION form.)
☐ Protocol meets the criteria for waiver of authorization. (Attach the HRP-901, WAIVER OF HIPAA AUTHORIZATION REQUEST form.)
☐ Protocol is using de-identified information. (Attach the HRP-902, DE-IDENTIFICATION CERTIFICATION form.) (Checked "None" in 1.0 above)
☐ Protocol involves research on decedents. (Attach the HRP-903, RESEARCH ON DECEDENTS REQUEST form.)
☐ Protocol is using a limited data set and data use agreement. (Contact the Office of Technology Commercialization to initiate a Limited Data Use Agreement.

***Find the HIPAA forms in the IRB Website Library, Templates.**

Attach the appropriate HIPAA form on the "Local Site Documents, #3. Other Documents", page of the application.

3.0 How long will identifying information on each participant be maintained?

6 years

4.0 Describe any plans to code identifiable information collected about each participant.

Data pulls will be done using the REDCap de-identify function.

5.0 Check each box that describes steps that will be taken to safeguard the confidentiality of information collected for this research:

- ✓ **Research records will be stored in a locked cabinet in a secure location**
- ✓ **Research records will be stored in a password-protected computer file**

The list linking the assigned code number to the individual subject will be maintained separately from the other research data

- ✓ **Only certified research personnel will be given access to identifiable subject information**

6.0 Describe the provisions included in the protocol to protect the privacy interests of subjects, where "privacy interests" refer to the interest of individuals in being left alone, limiting access to them, and limiting access to their information. (This is not the same provision to maintain the confidentiality of data.)

Trained study staff will protect the privacy by not gathering more data then needed and not reaching out to contact the family more than three times for recruitment and follow up surveys.

Confidential Health Information

1.0 Please mark all categories that reflect the nature of health information to be accessed and used as part of this research.

☒ Demographics (age, gender, educational level)
☒ Diagnosis
☒ Laboratory reports
☐ Radiology reports
☒ Discharge summaries
☒ Procedures/Treatments received
☒ Dates related to course of treatment (admission, surgery, discharge)
☐ Billing information
☒ Names of drugs and/or devices used as part of treatment
☒ Location of treatment
☐ Name of treatment provider
☒ Surgical reports
☒ Other information related to course of treatment
☐ None

2.0 Please discuss why it is necessary to access and review the health information noted in your response above.
Our study is gathering a complete history of each patient's pilonidal disease which includes the information above. This information will be used to identify the severity of treatments before and after laser/standard of care treatments.

3.0 Is the health information to be accessed and reviewed the minimal necessary to achieve the goals of this research? ☒ Yes ☐ No

4.0 Will it be necessary to record information of a sensitive nature? ☒ Yes ☐ No

5.0 Do you plan to obtain a federally-issued Certificate of Confidentiality as a means of protecting the confidentiality of the information collected? ☐ Yes ☒ No

8. Appendix:

Summary Sheet:

- Fitzpatrick Skin Classification Scale
- NRS-11 Pain Scale
- Child Attitude Toward Illness Scale (CATIS)
- Parent Stigma Scale
- Child Stigma Scale
- Sample of Study Data Collection Questionnaire
- PedsQL Healthcare Satisfcation Survey
- PedsQL HRQOL Survey examples:
    - PedsQL Parent Report for Teens (ages 13-18)
    - PedsQL Teen Report (ages 13-18)

Sample Measures:

Fitzpatrick Skin Classification System:

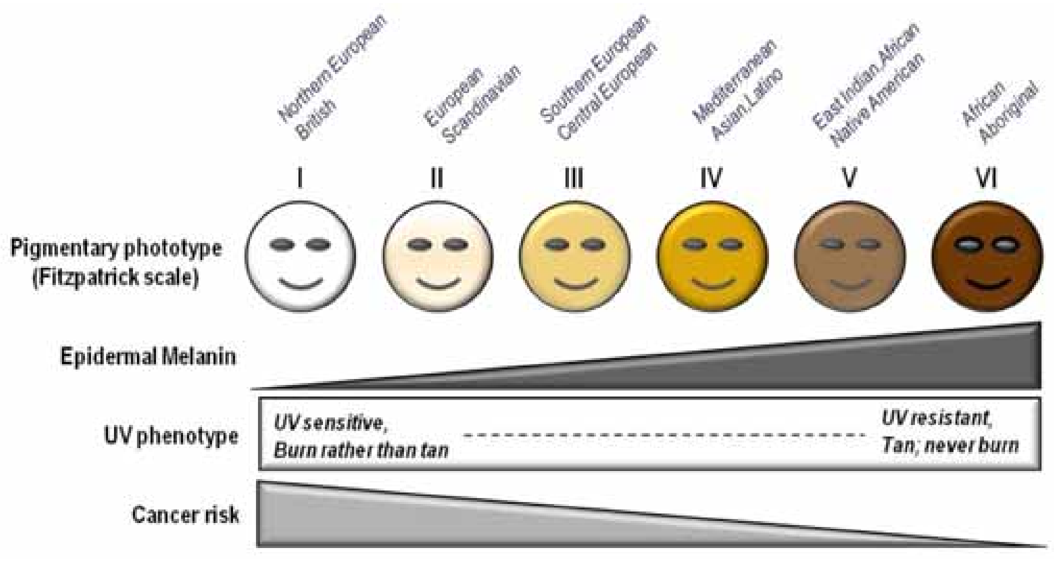

NRS-11 Pain Scale:

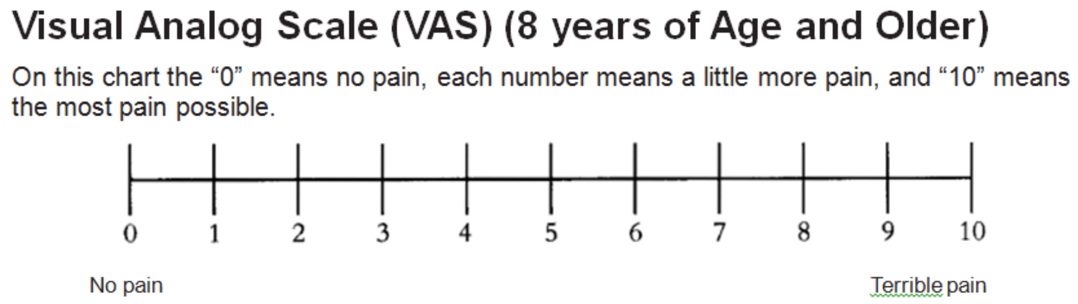

The Child Attitude Toward Illness Scale (CATIS):

1. How good or bad do you feel it is that you have pilonidal disease?
   (very good, a little good, not sure, a little bad, very bad)
2. How fair is it that you have pilonidal disease?
   (very fair, a little fair, not sure, a little unfair, very unfair)
3. How happy or sad is it for you to have pilonidal disease?
   (very sad, a little sad, not sure, a little happy, very happy)
4. How bad or good do you feel it is to have pilonidal disease?
   (very good, a little good, not sure, a little bad, very bad)
5. How often do you feel that you pilonidal disease is your fault?
   (never, not often, sometimes, often, very often)
6. How often do you feel that you pilonidal disease keeps you from doing things you like?
   (very often, often, sometimes, not often, never)
7. How often do you feel that you will always be sick?
   (never, not often, sometimes, often, very often)

8. How often do you feel that your pilonidal disease keeps you from starting new things?
   (very often, often, sometimes, not often, never)
9. How often do you feel different from others because of your pilonidal disease?
   (very often, often, sometimes, not often, never)
10. How often do you feel bad because you have pilonidal disease?
    (very often, often, sometimes, not often, never)
11. How often do you feel sad about being sick?
    (never, not often, sometimes, often, very often)
12. How often do you feel happy even though you have pilonidal disease?
    (never, not often, sometimes, often, very often)
13. How often do you feel just as good as other kids your age even though you have pilonidal disease?
    (very often, often, sometimes, not often, never)

Parent Stigma Scale:

Rating Scale: 1=strongly disagree; 2=disagree; 3=neither; 4=agree; 5=strongly agree

1. People who know that ______ has pilonidal disease treat him/her differently.
2. It really doesn't matter what I say to people about ______ pilonidal disease, they usually have their minds made up.
3. ________ always has to prove him/herself because of their pilonidal disease.
4. Because of the pilonidal disease, __________will have problems finding a husband or wife.
5. In many people's minds, having pilonidal disease attaches a stigma or label to ________

Child Stigma Scale:

Rating Scale: 1=never; 2=not often; 3=sometimes; 4=often; 5=very often

1. How often do you feel different form other kids because you have pilonidal disease?
2. How often do you feel people may not like you if they know you have pilonidal disease?
3. How often do you feel other children are uncomfortable with you because of your pilonidal disease?
4. How often do you feel people may not want to be friends with you if they know you have pilonidal disease?
5. How often do you feel people would not want to go out with you or ask you to parties if they now you have pilonidal disease?
6. How often do you feel embarrassed about your pilonidal disease?
7. How often do you keep your pilonidal disease a secret from other kids?
8. How often do you try to avoid talking to other people about your pilonidal disease?

Sample Study Data Collection Questionnaire: Pilonidal Management (Child):

The items below will be modified to reflect the appropriate time point in the study
For baseline: wording will be modified to say, in the last month…
For the treatment phase: wording will be modified to say, since you last treatment…
For the follow-up time points: wording will be modified to say, since your last follow-up…

1. In the last month, how many days did you experience pain due to your pilonidal disease?
   __________ number of days
2. When you experienced pain, can you tell me how severe the pain was on a scale from 1-10, 1 being no pain and 10 being terrible pain
3. On average, how long would your pain last?
   a. Minutes
   b. Hours
   c. Days
   d. The entire time, I am still in pain
4. Since your last treatment have you experienced any of the following symptoms?:
   - Swelling, redness, or sore skin in and around the treatment area yes/no
     If yes, did you see a doctor for these symptoms y/n
     If yes, please describe the instructions and treatments that the doctor provided
   - Pus or blood (drainage of pus or blood) in or around the treatment area yes/no
     If yes, did you see a doctor for these symptoms y/n
     If yes, please describe the instructions and treatments that the doctor provided
   - A bad odor coming from the treatment area yes/no
     If yes, did you see a doctor for these symptoms y/n
     If yes, please describe the instructions and treatments that the doctor provided
   - Holes or openings in or around the treatment area yes/no
     If yes, did you see a doctor for these symptoms y/n
     If yes, please describe the instructions and treatments that the doctor provided
5. Since your last treatment did you use nair or shaving for your pilonidal disease?
   - Yes, I used nair only
   - Yes, I used shaving only
   - Yes, I used both nair and shaving
   - No, I did not use either
6. Since your last treatment how often have you used nair (branch from 1 or 3)?
   - More than 1 time per week
   - 1 time per week
   - At least 2 times since the last treatment
   - At least 1 time since the last treatment
   - I did not use the nair at all since my last treatment
7. Did someone help you with applying the nair to the treatment area?
   - Yes
   - No
8. Since your last treatment how often have you shaved (branch from 2 or 3)?

- More than 1 time per week
- 1 time per week
- At least 2 times since the last treatment
- At least 1 time since the last treatment
- I did not shave at all since my last treatment

9. Did someone help you with shaving the treatment area?
   - Yes
   - No
10. Since your last treatment has your pilonidal disease caused you to have trouble or discomfort with any of the activities below?
    - Bathing or keeping yourself clean   Yes/No
      - If yes, did you need help from another person with this activity? Yes/No
    - Shaving or applying nair to your treatment area   Yes/No
      - If yes, did you need help from another person with this activity? Yes/No
    - Going to the bathroom Yes/No
      - If yes, did you need help from another person with this activity? Yes/No
    - Dressing yourself   Yes/No
      - If yes, did you need help from another person with this activity? Yes/No
    - Comfortably sitting still or laying down Yes/No
      - If yes, did you need help from another person with this activity? Yes/No
    - Walking around the house, school, or work Yes/No

If yes, did you need help from another person with this activity? Yes/No

PedsQL Healthcare Satisfaction:

How happy are you with…

INFORMATION	Never	Some-times	Often	Almost Always	Always	Not Applicable
1. How much information was provided to you about your child's diagnosis	0	1	2	3	4	N/A
2. How much information was provided to you about the treatment and course of your child's health condition	0	1	2	3	4	N/A
3. How much information was provided to you about the side effects of your child's treatment	0	1	2	3	4	N/A
4. How soon information was given to you about your child's test results	0	1	2	3	4	N/A
5. How often you are updated about your child's health	0	1	2	3	4	N/A

INCLUSION OF FAMILY	Never	Some-times	Often	Almost Always	Always	Not Applicable
1. The sensitivity shown to you and your family during your child's treatment	0	1	2	3	4	N/A
2. The willingness to answer questions that you and your family may have	0	1	2	3	4	N/A
3. The effort to include your family in discussion of your child's care and other information about your child's health condition	0	1	2	3	4	N/A
4. How much time the staff gave you to ask any questions you may have had about your child's health condition and treatment	0	1	2	3	4	N/A

**COMMUNICATION**	**Never**	**Some-times**	**Often**	**Almost Always**	**Always**	**Not Applicable**
1. How well the staff explained your child's health condition and treatment to **your child** in a way that she/he could understand	0	1	2	3	4	N/A
2. The time taken to explain your child's health condition and treatment to **you** in a way you could understand	0	1	2	3	4	N/A
3. How well the staff listens to you and your concerns	0	1	2	3	4	N/A
4. The preparation provided for **you** about what to expect during tests and procedures	0	1	2	3	4	N/A
5. The preparation provided for **your child** about what to expect during tests and procedures	0	1	2	3	4	N/A

**TECHNICAL SKILLS**	**Never**	**Some-times**	**Often**	**Almost Always**	**Always**	**Not Applicable**
1. How well the staff responds to your child's needs	0	1	2	3	4	N/A
2. Efforts to keep your child comfortable and as pain-free as possible	0	1	2	3	4	N/A
3. How much time the staff took to help you with your child coming back home	0	1	2	3	4	N/A

**EMOTIONAL NEEDS**	**Never**	**Some-times**	**Often**	**Almost Always**	**Always**	**Not Applicable**
1. The amount of time given to your child to play, talk about her/his feelings, and any questions she/he may have	0	1	2	3	4	N/A

2. The amount of time spent helping your child with going back to school	0	1	2	3	4	N/A
3. The amount of time spent attending to **your child's** emotional needs	0	1	2	3	4	N/A
4. The amount of time spent attending to **your** emotional needs	0	1	2	3	4	N/A

**OVERALL SATISFACTION**	**Never**	**Some-times**	**Often**	**Almost Always**	**Always**	**Not Applicable**
1. The overall care your child is receiving	0	1	2	3	4	N/A
2. How friendly and helpful the staff is	0	1	2	3	4	N/A
3. The way your child is treated at the hospital	0	1	2	3	4	N/A

PedsQL Parent Report for Teens (ages 13-18):

*In the past **ONE month,** how much of a **problem** has your teen had with …*

**Physical Functioning** ***(PROBLEMS WITH…)***	***Never***	**Almost Never**	***Some-times***	**Often**	***Almost Always***
1. Walking more than one block	0	1	2	3	4
2. Running	0	1	2	3	4
3. Participating in sports activity or exercise	0	1	2	3	4
4. Lifting something heavy	0	1	2	3	4
5. Taking a bath or shower by him or herself	0	1	2	3	4
6. Doing chores around the house	0	1	2	3	4
7. Having hurts or aches	0	1	2	3	4
8. Low energy level	0	1	2	3	4
**Emotional Functioning** ***(PROBLEMS WITH…)***	***Never***	**Almost Never**	***Some-times***	**Often**	***Almost Always***
1. Feeling afraid or scared	0	1	2	3	4
2. Feeling sad or blue	0	1	2	3	4
3. Feeling angry	0	1	2	3	4
4. Trouble sleeping	0	1	2	3	4
5. Worrying about what will happen to him or her	0	1	2	3	4
Social Functioning **(PROBLEMS WITH…)**	***Never***	**Almost Never**	***Some-times***	**Often**	***Almost Always***
1. Getting along with other teens	0	1	2	3	4
2. Other teens not wanting to be his or her friend	0	1	2	3	4

3. Getting teased by other teens	0	1	2	3	4
4. Not able to do things that other teens his or her age can do	0	1	2	3	4
5. Keeping up with other teens	0	1	2	3	4
School Functioning **(PROBLEMS WITH…)**	***Never***	**Almost Never**	***Some-times***	**Often**	***Almost Always***
1. Paying attention in class	0	1	2	3	4
2. Forgetting things	0	1	2	3	4
3. Keeping up with schoolwork	0	1	2	3	4
4. Missing school because of not feeling well	0	1	2	3	4
5. Missing school to go to the doctor or hospital	0	1	2	3	4

PedsQL Teen Report (ages 13-18)

*In the past **ONE month,** how much of a **problem** has your teen had with …*

*In the past **ONE month**, how much of a **problem** has this been for you …*

**About My Health and Activities** ***(PROBLEMS WITH…)***	***Never***	**Almost Never**	***Some-times***	**Often**	***Almost Always***
1. It is hard for me to walk more than one block	0	1	2	3	4
2. It is hard for me to run	0	1	2	3	4
3. It is hard for me to do sports activity or exercise	0	1	2	3	4
4. It is hard for me to lift something heavy	0	1	2	3	4
5. It is hard for me to take a bath or shower by myself	0	1	2	3	4
6. It is hard for me to do chores around the house	0	1	2	3	4
7. I hurt or ache	0	1	2	3	4
8. I have low energy	0	1	2	3	4
**About My Feelings** ***(PROBLEMS WITH…)***	***Never***	**Almost Never**	***Some-times***	**Often**	***Almost Always***
1. I feel afraid or scared	0	1	2	3	4
2. I feel sad or blue	0	1	2	3	4
3. I feel angry	0	1	2	3	4
4. I have trouble sleeping	0	1	2	3	4
5. I worry about what will happen to me	0	1	2	3	4
**How I Get Along with Others** ***(PROBLEMS WITH…)***	***Never***	**Almost Never**	***Some-times***	**Often**	***Almost Always***
1. I have trouble getting along with other teens	0	1	2	3	4
2. Other teens do not want to be my friend	0	1	2	3	4

3. Other teens tease me	0	1	2	3	4
4. I cannot do things that other teens my age can do	0	1	2	3	4
5. It is hard to keep up with my peers	0	1	2	3	4
**About School *(PROBLEMS WITH…)***	***Never***	**Almost Never**	***Some-times***	**Often**	***Almost Always***
1. It is hard to pay attention in class	0	1	2	3	4
2. I forget things	0	1	2	3	4
3. I have trouble keeping up with my schoolwork	0	1	2	3	4
4. I miss school because of not feeling well	0	1	2	3	4
5. I miss school to go to the doctor or hospital	0	1	2	3	4

OSU MyChart Messaging

A randomized controlled trial of laser depilation in the treatment of pilonidal disease.

Several studies have found that laser hair removal reduces the recurrence rate of pilonidal disease. Drs. Minneci and Deans, pediatric surgeons in the Ohio State University Department Surgery, are currently conducting a study that will help better understand how laser hair removal can help patients, 11-21 years old, with pilonidal disease.

Based on your recent visit(s) to the Ohio State University Health Care System for treatment of pilonidal disease, you may qualify for a study that is currently being conducted at Nationwide Children's Hospital entitled: Randomized Controlled Trial of Laser Hair Depilation in Adolescents and Young Adults with Pilonidal Disease.

Participants of the study will be assigned to receive laser hair removal or standard care. All participants will be followed for two years, with follow-ups occurring, at 1, 2, 3, 4, 5, 6, 9, 12, 18, and 24 months after enrollment. Compensation is available at certain follow-up time points and laser hair removal is free-of-charge.

Eligibility criteria:

- Between the ages of 11 - 21 years old
- Previous diagnosis and treatment for pilonidal disease

If you are interested in this study and are under 18 years of age, then we will also need to discuss this study with your parent or legal guardian.
